# Multimodal bHLH-PAS DNA binding controls specificity and drives obesity

**DOI:** 10.1093/nar/gkaf1352

**Published:** 2026-01-06

**Authors:** David C Bersten, Daniel P McDougal, Adrienne E Sullivan, Alexis Gerassimou, James Breen, Rebecca L Fitzsimmons, George E O Muscat, Stephen Pederson, John B Bruning, Chen-Ming Fan, Paul Q Thomas, Darryl L Russell, Daniel J Peet, Murray L Whitelaw

**Affiliations:** The Department of Molecular and Biomedical Science, School of Biological Sciences, The University of Adelaide, Adelaide, SA 5001, Australia; Robinson Research Institute, School of Biomedicine, The University of Adelaide, Adelaide, SA 5001, Australia; The Department of Molecular and Biomedical Science, School of Biological Sciences, The University of Adelaide, Adelaide, SA 5001, Australia; Institute for Photonics and Advanced Sensing, The University of Adelaide, Adelaide, SA 5001, Australia; The Department of Molecular and Biomedical Science, School of Biological Sciences, The University of Adelaide, Adelaide, SA 5001, Australia; Adelaide Centre for Epigenetics, School of Biomedicine, Faculty of Health and Medical Sciences, The University of Adelaide, Adelaide, SA 5001, Australia; South Australian immunoGENomics Cancer Institute, Faculty of Health and Medical Sciences, The University of Adelaide, Adelaide, SA 5001, Australia; The Department of Molecular and Biomedical Science, School of Biological Sciences, The University of Adelaide, Adelaide, SA 5001, Australia; Robinson Research Institute, School of Biomedicine, The University of Adelaide, Adelaide, SA 5001, Australia; Bioinformatics Hub, University of Adelaide, Adelaide, SA 5001, Australia; Institute for Molecular Bioscience, University of Queensland, Brisbane, QLD 4067, Australia; Institute for Molecular Bioscience, University of Queensland, Brisbane, QLD 4067, Australia; Bioinformatics Hub, University of Adelaide, Adelaide, SA 5001, Australia; The Department of Molecular and Biomedical Science, School of Biological Sciences, The University of Adelaide, Adelaide, SA 5001, Australia; Institute for Photonics and Advanced Sensing, The University of Adelaide, Adelaide, SA 5001, Australia; Carnegie Institution for Science, Department of Embryology, Baltimore, MD 21218, United States; Gene Editing Program, Precision Medicine Theme, South Australian Health and Medical Research Institute, Adelaide, SA 5001, Australia; Robinson Research Institute, School of Biomedicine, The University of Adelaide, Adelaide, SA 5001, Australia; The Department of Molecular and Biomedical Science, School of Biological Sciences, The University of Adelaide, Adelaide, SA 5001, Australia; The Department of Molecular and Biomedical Science, School of Biological Sciences, The University of Adelaide, Adelaide, SA 5001, Australia; ASEAN Microbiome Nutrition Centre, National Neuroscience Institute, Singapore 169857, Singapore

## Abstract

The basic-helix-loop-helix Per-Arnt-Sim (PAS) homology domain (bHLH-PAS) transcription factor (TF) family comprises critical sensors or actuators of physiological (hypoxia, tryptophan metabolites, neuronal activity, and appetite) and environmental (diet-derived metabolites and pollutants) stimuli regulating genes involved in signal adaptation and homeostasis. Despite the importance of this TF family, the mechanisms underlying specificity of DNA binding and target gene regulation by the bHLH-PAS subfamily remain unresolved. We systematically analysed cognate DNA binding hierarchies of prototypical bHLH-PAS family members (ARNT, ARNT2, HIF1α, HIF2α, AhR, NPAS4, SIM1), revealing large DNA binding footprints (12–15 bp) and unique mechanisms of DNA binding specificity involving preferential DNA sequences flanking the core motif. Flank-encoded DNA binding specificity discerns otherwise identical core sequence binding by SIM1 and the HIFs, mediated through N-terminal HIFα–DNA interactions. We also reveal an intimate relationship between DNA shape and core and flank TF binding that allows motif sequence flexibility and underpins multimodal mechanisms for achieving TF binding specificity. Furthermore, novel downstream SIM1 PAS-loop/DNA interactions are associated with AT-rich sequences contributing to DNA binding and transcriptional activity; these interactions are critical for TF biological function underpinning a monogenic cause of human hyperphagic obesity in a recapitulated SIM1.R171H knock-in mouse model.

## Graphical Abstract

**Graphical Abstract:**
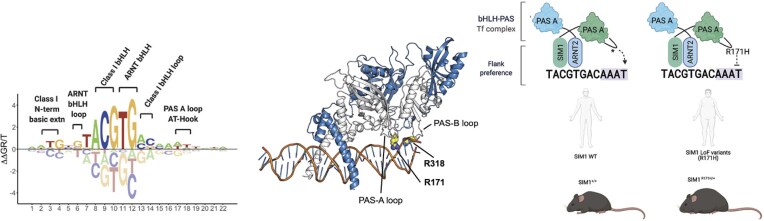

## Introduction

Gene regulation is mediated by DNA-binding transcription factors (TFs) via distinct DNA response elements. While consensus DNA sequences play a key role in determining genome occupancy and target gene selection *in vivo*, multiple additional mechanisms must contribute to specificity of TF binding [1–4].

The bHLH-PAS TF family represents an important model to investigate the mechanisms underlying TF specificity, as the family members bind both shared and distinct response elements [5], display cell-type-specific chromatin occupancy and gene expression [6–8], and perform distinct biological processes. For example, the hypoxic-inducible factors (HIF1α and HIF2α) display non-overlapping biological roles, cell-type- or isoform-specific (HIF1α versus HIF2α) chromatin occupancy, and target gene regulation by unresolved mechanisms [6, 9]. In addition, NPAS4 displays promiscuous response element DNA binding [8, 10] and performs opposing roles in inhibitory and excitatory neurons via target gene regulation to collectively control neuronal network activity. SIM1, SIM2, NPAS1, NPAS3, HIF1α, and HIF2α also all recognize RCGTG (R = A/G) core consensus sequences but perform distinct biological roles in appetite control and hypothalamic development [5] (SIM1 and SIM2), inhibitory neuron development and activity [11] (NPAS1 and NPAS3), and the hypoxic response [6] (HIF1α and HIF2α), indicating that additional encoded specificity is yet to be deciphered.

bHLH-PAS TFs also uniquely bind NNCGTG (CACG–E-box-like) DNA sequences as heterodimers, containing one Class I (HIF1α, HIF2α, AhR, SIM1, NPAS1, NPAS3, NPAS4) protein with one Class II (ARNT or ARNT2) partner. The PAS domains restrict heterodimeric partner selection to within the bHLH-PAS subfamily and strengthen DNA binding and heterodimerization. PAS domains have also been shown to mediate protein–protein interaction with co-activators or other TFs, and PAS domain swap experiments in *Drosophila* homologs is sufficient to switch target gene expression [12].

However, whether specific, sequence-dependent interactions between the PAS domains and DNA outside the core motifs have a pivotal role in mediating bHLH-PAS TF site selection and hence target gene regulation *in vivo* remains to be defined. To answer this major outstanding question regarding the function of this family of biologically critical TFs, this study profiled bHLH-PAS TF members using high-throughput DNA binding assays and coupled computational analyses to determine the factors underlying their inherent DNA binding specificities.

Through analysis of inherent bHLH-PAS heterodimer response element specificity, *in vivo* chromatin occupancy and DNA methylation patterns, DNA shape-affinity relationships, protein–DNA structural analysis, and a novel Sim1^R171H^ knock-in mouse model of obesity, we reveal mechanisms underlying target specificity of the bHLH-PAS TF family. We demonstrate that bHLH-PAS TFs bind to a much larger footprint (12–15 bp) than previously appreciated and that TF–DNA contacts outside the core motif are important specificity, strength and transcriptional activity. Moreover, using a mouse model of the human obesity variant SIM1.R171H, we demonstrate that novel PAS domain binding outside of the core motif is also critical for biological function and mechanistically underpins this case of hyperphagic obesity.

## Materials and methods

### Purification of bHLH-PAS heterodimers

Truncated bHLH-PAS constructs were cloned into MultiBAC baculoviral transfer plasmid pFBDM [13] with a 6×His-TEV leader with isothermal assembly [14], such that each construct expressed a Class I tagged TF and an untagged ARNT or ARNT2 from a single baculovirus. pEFIRESpuro-hARNT-3×Flag and pEFIRESpuro-hARNT2-3×Flag, pET16b-6×His-TEV-hAhR(1–287) (AmpR) and pAC28-hARNT(1–362) were described previously [15, 16]. 

### Baculoviral expression and purification

MultiBAC-LoxP-EYFP was generated by recombination of pUDCM-EYFP into the MultiBAC genome by Cre transposition as described [13]. With the exception of NPAS4, all truncated bHLH-PAS (A and B) heterodimers were incorporated into the MultiBAC-LoxP-EYFP baculoviral genome by pFBDM Tn7 transposition as described [13, 17]. Truncated mNPAS4 (1–329)/mARNT2 (1–481)/mnucTomato (monomeric nuclear) were recombined into MultiBAC by Tn7 transposition. Baculovirus was produced by transfection into Sf9 cells cultured in SF900III media as described [17], and protein production monitored by eYFP or nucTomato fluorescence microscopy.

### Bacterial AhR expression and purification

BL21(DE3) (LysS) bacterial cells were co-transformed with pET16b-6×His-TEV-hAhR(1–287) (AmpR)/pAC28-hARNT(1–362) (KanR). Bacteria were grown in Luria Broth to an OD600 of 0.6, and protein expression was induced by the addition of 1 mM Isopropyl β-D-thiogalactoside (IPTG) at 16°C for 18 h.

Insect- or bacterially expressed proteins were purified by His-tag purification using HiTrap FF columns (GE), HisPur Resin (Thermo Scientific), or HisPur Cobalt Resin (Thermo), followed by His-tag removal by Tobacco etch virus (TEV) protease (in-house generated) cleavage, ion-exchange or heparin affinity chromatography, and/or size-exclusion chromatography. Cell pellets were lysed by sonication (15 s ON, 30 s OFF for 3 min; insect or bacterial TEVp) or high-pressure mechanical disruption (bacteria AhR/ARNT) in a buffer containing 20 mM Tris pH 8.0 (or Hepes), 500 mM NaCl, 15 mM imidazole, 5 mM β–mercaptoethanol, 10% glycerol, 0.2% Triton X-100, 0.5× ethylenediaminetetraacetic acid (EDTA) free protease inhibitors (Roche Cat # 04693132001 cOmplete, EDTA-free protease inhibitor tablets). Lysates were clarified 1–2× by centrifugation at 40–50 000 *g* for ∼45 min and filtered through a 0.45 μM cellulose acetate filter prior to batch or column purification. Column purifications were performed using an NGC Discover FPLC system (Bio-Rad) and used a gradient of imidazole (generally 15–250 mM) over ∼20 column volumes after loading and washing with lysis buffer and Buffer A [20 mM Tris, pH 8.0 (or Hepes), ∼500 mM NaCl, 15–40 mM imidazole, 5 mM β–mercaptoethanol, 10% glycerol]. HisPur batch purification was performed in gravity columns, where lysate was passed over the column and the beads were washed with 10–20 bed volumes of lysis buffer, followed by Buffer A [20 mM Tris, pH 8.0 (or Hepes), ∼500 mM NaCl, 15–40 mM imidazole, 5 mM β–mercaptoethanol, 10% glycerol] and elution with Buffer B [20 mM Tris, pH 8.0 (or Hepes), 500 mM NaCl, 250–500 mM imidazole, 5 mM β–mercaptoethanol, 10% glycerol]. Purified protein was then buffer exchanged by dialysis or 10 kDa MWCO filter into 20 mM Tris–HCl, pH 8.0 (or 20 mM Hepes pH 8.0), 15 mM imidazole, 300 mM NaCl, 5 mM β–mercaptoethanol, and 10% glycerol. 6×His-TEV protease was added at a ratio of 1:5–20 and incubated overnight at 4°C to remove the 6×His tag. If necessary, the protein was further buffer exchanged prior to passing over a His column or beads to remove the 6×His-TEV. Protein was then diluted to ∼75 mM NaCl, giving a final buffer of 20 mM Hepes pH 8.0, 75 mM NaCl, 5%–10% glycerol, and 10 mM Dithiothreitol (DTT). Protein was further purified by ion-exchange chromatography or heparin affinity chromatography with a gradient of 75–1000 mM NaCl. If required, size-exclusion chromatography using a buffer of (20 mM Hepes, pH 8.0, 300 mM NaCl, 5%–10% glycerol, 10 mM DTT) was performed to further purify the protein. Protein purity was assessed by sodium dodecyl sulphate–polyacrylamide gel electrophoresis (SDS–PAGE) and Coomassie staining, and concentrations estimated by A280 absorbance. Purified proteins were stored in a buffer containing 20 mM Hepes, pH 8.0, 300 mM NaCl, 5%–10% glycerol, 10 mM DTT and flash-frozen in LiN_2_ for long term storage at −80°C. This approach provided untagged heterodimeric bHLH-PAS TF complexes.

For Expi293 mammalian expression and purification of C-terminal 3×Flag-tagged full-length ARNT or ARNT2 plasmids (pEFIRES-Puro-hARNT-3×Flag or pEFIRES-Puro-hARNT2-3×Flag) were transiently transfected (200 μg) into 100 ml of 1.5 × 10^6^ cell/ml Expi293 cells (Life Technologies) grown in Expi293SF media using PEI polyethylenimine (PolySciences). Approximately 60 h post-transfection, Expi293 cells were harvested by centrifugation, washed with phosphate buffered saline (PBS), and lysed in 4 ml of 20 mM Hepes (pH 8.0), 500 mM NaCl, 1% Triton X-100, 1 mM EDTA, 2× protease inhibitors, 2 mM NaVO_4_, 10 mM β–glycerophosphate, 10 mM NaF, 10% glycerol. Lysate was incubated with 75 μl of Flag M2 O/N at 4°C end-on-end. The Flag M2 resin was then washed with 5 × 1 ml lysis buffer, 2 × 1 ml 0.1% CHAPS wash buffer (20 mM Hepes, pH 8.0, 0.1% CHAPS, 250 mM NaCl, 1 mM EDTA, 5% glycerol), 2 × 1 ml 0.02% NP-40 wash buffer (20 mM Hepes, pH 8.0, 0.02% NP-40, 250 mM NaCl, 1 mM EDTA, 5% glycerol), and eluted with 150 μl of NP-40 wash buffer +250 ng/ml 3×Flag peptide end-on-end 1 h incubation at 4°C. Purified ARNT-3×Flag or ARNT2-3×Flag homodimers were then enriched using 100 kDa MWCO filters (Ambion), while removing some contaminants. The concentration and purity of ARNT-3×Flag or ARNT2-3Flag was estimated by SDS–PAGE and Coomassie staining with bovine serum albumin (BSA) standards.

### SELEX-seq

Two hundred fifty nanomolar of purified TF and 200 nM [1.5 μl of 5 μM of DNA library (Random 18mer or FixedCore 18/22mer), Supplementary Table S1] of Fam-labelled Round 0 library were incubated at room temperature in buffer containing 20 mM Tris–HCl, pH 8.0, 3 mM MgCl_2_, 200–300 mM NaCl, 8% glycerol, 50 μg/ml polydI-dC, 0.2 mg/ml BSA, 5 mM β–mercaptoethanol in 30 μl for 20 min. DNA was extracted and amplified as described in [18]. DNA isolated from SELEX Round 0 (initial library), Round 1 and Round 2 (FixedCore 8NCGTG10N), or Round 3 (Random 18mer) were barcoded as described in [18], and Illumina-compatible adapters added by limited-cycle polymerase chain reaction (PCR) and cleaned up by PAGE purification. FixedCore 18/22mer SELEX samples from each round (Round 0, Round 1, Round 2) were quantified, pooled, and run on separate lanes of a Hiseq2500 run. Random 18mer SELEX samples (Round 0 and Round 3) were barcoded by limited-cycle PCR, quantified, pooled, and sequenced on a NEXTSeq500 using a single end 1 × 75 bp High Output mode, resulting in 20–30 million reads per sample. Oligonucleotides used for SELEX-seq and electrophoretic mobility shift assay (EMSA) experiments are available in Supplementary Table S1. The protein truncations and SELEX experiments performed in this manuscript are described in Supplementary Table S2.

### TF DNA binding models

Base-calling and demultiplexing was achieved with bcl2fastq, fastq files were quality filtered using Fastx toolkit [19]. In some analysis, k-mer (a subsequence of nucleotides) enrichment and TF binding models were generated with filtered fastq data to remove more than one “Core” (CGTG) binding site per read. One Core motif per read was filtered using fastq-tools (https://github.com/dcjones/fastq-tools, version 0.8) fast-grep -v function. Kmer counting and relative enrichments were analysed using the SELEX-seq R package [18, 20] (version 1.2), TF binding motif models generated using No Read Left Behind (NRLB) [21] https://github.com/BussemakerLab/NRLB and ProBound [22], run on The University of Adelaide Phoenix High Performance Computing node. Demultiplexing and trimming of primers and barcode sequences were removed using SELEX-seq or NRLB during analysis by specifying flanks and barcodes. MAX SELEX data from PRJEB25690 EBI [21] was also filtered for one core per line prior to re-analysis with SELEX-seq and NRLB to minimize multiple binding events per sequence. NRLB models were used to score BED using a custom R script modified from NRLBtools. Probound and NRLB models and parameters are provided in Supplementary Data File 1.

### EMSA and methylC EMSA

EMSAs were performed with different competitor DNA [single-stranded DNA (ssDNA)–salmon sperm DNA] conditions than SELEX-seq using Fam-lablled double-stranded DNA (dsDNA) probes, generated by annealing upper fam labelled Oligonucleotides (IDT DNA, Supplementary Table S1). Briefly, protein–DNA complexes were formed *in vitro* by incubating increasing amounts of TF (0–5 μg) with 10 nM of EMSA probes in a buffer containing 20 mM Tris, pH 8.0, 250 mM NaCl, 16 μg/ml ssDNA, 30 μg/ml BSA, 1.25 mM MgCl_2_, 6% glycerol, 10 mM DTT. TF DNA complexes were incubated at room temperature for 30 min before separation of bound complexes by non-denaturing 5%–7% PAGE. Gels were scanned using ChemiDoc (Bio-Rad) with the fluorescein channel and bands intensity estimated using the Image Lab software (Bio-Rad). Relative binding of protein to different DNA probes was estimated by fraction of probe bound at a constant sub-saturating protein concentration across *n* = 3 independent experiments.

### SIM1 WT versus R171H *in vitro* DNA binding

For SIM1/ARNT2 protein expression and purification, hSIM1 (1–348) or SIM1.R171H (1–348) and hARNT2 (69–438) were cloned into a single co-expressing baculovirus vector pFBDM and transferred into MultiBac-mcherry bacmid by transformation of Tn7-expressing DH10EMBacVSVg cells. 12.5 μg of isolated bacmid DNA was transfected into 25 ml ExpiSf9 cells (Thermo) at 2.5 × 10^6^ cells/ml using 30 μl of ExpiFectamine Sf in 1ml of Optimem. V_0_ virus was harvested 3–5 days after transfection, and virus amplified by two rounds of infection to generate V_2_ virus. Twenty-five to fifty millilitres of V_2_ was used to infect ∼1–1.5 l of ExpiSf9 cells at ∼3–4 × 10^6^ cells/ml, and cells were harvested ∼3 days post-infection by centrifugation at 1500 rpm for 10 min when mCherry fluorescence had peaked (∼72 h) and snap frozen in LiN_2_. Protein was purified as described earlier with a minor addition of 1.2 units/ml of benzonase nuclease to lysis buffers.

## Comparative analysis of transcription factor DNA binding models

### NRLB and SELEX-seq affinity comparisons

**Figure 1.**
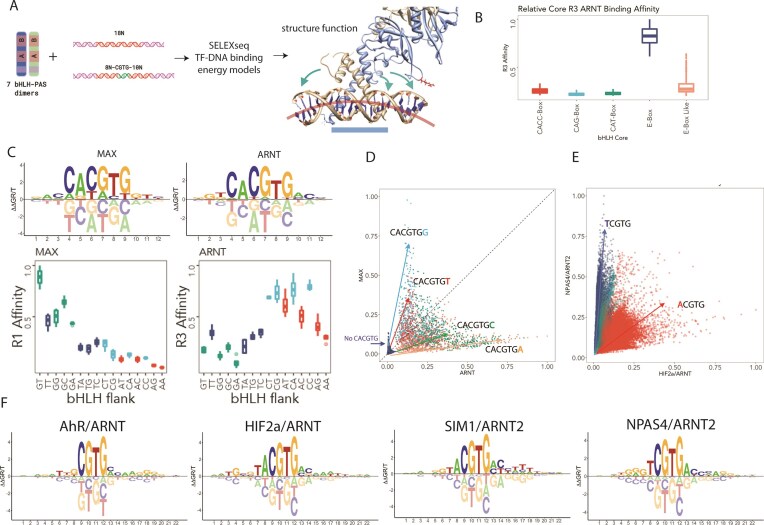
Inherent bHLH-PAS DNA binding affinity and specificity by SELEX-seq. (**A**) bHLH-PAS TF dimers were incubated with either random 18mer or FixedCore 8N-CGTG-10N dsDNA ligands prior to selection of bound DNA by EMSA, barcoding, and high-throughput sequencing. SELEX high-throughput sequencing was analysed using a combination of SELEX-seq R, NRLB protein–DNA modelling, and DNA shape, and compared to known (shown, HIF2α/ARNT/HRE 4ZPK) and modelled DNA-bound bHLH-PAS heterodimeric structures. (**B**) Round 3 10-mer affinity boxplots (R3 Affinity) of ARNT-mediated selection of E-box (CACGTG)-containing probes versus other known bHLH motifs (CACC-box (CACCTG), CAG-box (CAGCTG), CAT-box (CATATG), or E-box-like (DNCGTG; D = A, T, G)]. (**C**) MAX versus ARNT-derived 12-mer energy models (upper panel) reveal E-box flanking specificity, which is also observed in 10-mer affinity boxplots (affinity of palindromic CACGTGNN; f_+1_f_+2_ Kmers) (lower panel) (**D**) DNA binding specificity between bHLH subgroups ARNT versus MAX is encoded by E-box flanking sequences. Affinities of all NNNCANNTGNNN 12-mers were scored using TF–DNA binding model. Flanking sequences (CANNTGN; f_+1_) (or non-CACGTG containing sequences) are colour-coded as indicated. (**E**) bHLH-PAS TF heterodimer DNA binding can be distinguished by a single nucleotide upstream (NNCGTG; c_2_) of the E-box like core NCGTG. 12-mers from SELEX-seq were colour coded for NCGTG sequence as indicated. (**F**) Energy logos of TF–DNA binding models.

**Figure 2.**
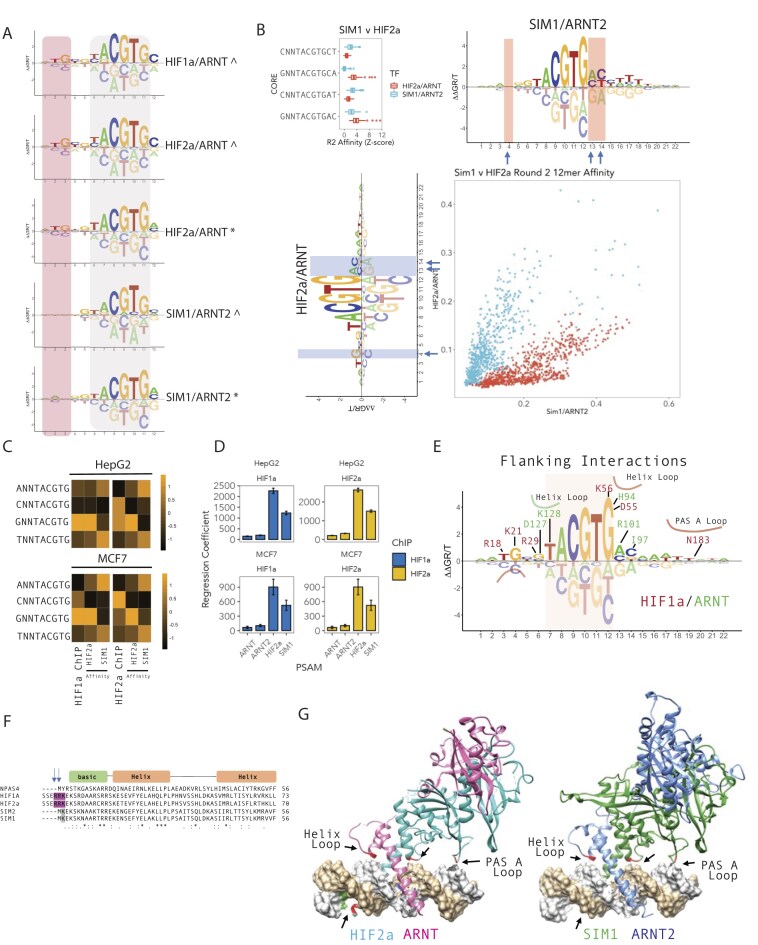
bHLH-PAS DNA binding specificity is encoded by nucleotides flanking the NNCGTG core. (**A**) Upstream nucleotide specificity of SIM1 versus HIFα (HIF1α or HIF2α) from SELEX-derived DNA binding energy models of HIFα/ARNT versus SIM1/ARNT2 from either random 18-mer (^) or FixedCore 18/22mer (*) SELEX strategies showed that HIFα subunits prefer T and G at positions f_-3_ and f_-4_ [upstream (shaded pink) of core NNCGTG (shaded grey)] versus C and C at positions f-3 and f-4. SIM1 SELEX did not appear to have a strong preference or aversion to nucleotides at positions f-3 and f-4. (**B**) Upstream f-3 (nucleotide −3 from NNCGTG) and downstream f+1–f+2 (nucleotides +1 and +2 from NNCGTG) encode specificity between shared core-binding (TACGTG) SIM1/ARNT2 and HIF2α/ARNT TFs. Scatterplot comparing k-mer affinities (a subset of 12-mers) for HIF2α/ARNT versus SIM1/ARNT2, coloured by blue = GNNNACGTG or red = CNNNACGTG, demonstrating preferential DNA selection by SIM1/ARNT2 or HIF2α/ARNT. (Top left) Boxplots comparing selective round 2 12-mer k-mer affinities (Z-score normalized). Blue arrows indicate most variant positions in energy logos between HIF2α/ARNT and SIM1/ARNT2. (**C**) Upstream f-3 HIFα preferential sequence specificity is found at HIFα ChIP-seq peaks in HepG2 (upper panel) and MCF7 cells (lower panel). ChIP-seq DNA regions were scored by NRLB energy models for SIM1/ARNT2 and HIF2α/ARNT, and mean scores were compared to ChIP-seq peak scores at HIF1α/HIF2α peaks and represented by heatmap. (**D**) Linear regression coefficients (±SE) comparing models of *in vitro* DNA binding to *in vivo* ChIP-seq data. HIF1α (blue) or HIF2α (yellow) ChIP-seq peak DNA (HepG2 or MCF7) was scored using NRLB models for HIF2α/ARNT, SIM1/ARNT2, ARNT, or ARNT2. (**E**) Extensive protein–DNA contacts contribute to core (NNCGTG, shaded orange) flanking nucleotide specificity. Overlay of HIF2α/ARNT energy model with HIF2α (red) or ARNT (green) amino acid–DNA (phosphate, sugar, or base) contacts curated from PDB 4ZPK. Basic, helix or PAS-A loop domain interactions are outlined. (**F**) Basic amino acids in an N-terminal extension of HIF1α and HIF2α, not observed in SIM1 or SIM2, interact with upstream (f-3 and f-4 from NNCGTG) sites. Blue arrows indicate positions in HIFα that contact the *T* and *G* at positions −3 and −4 (upstream of core NNCGTG). (**G**) Structures of HIF1α/ARNT and SIM1/ARNT2 on DNA were used to illustrate protein–DNA interactions that contribute to flanking nucleotide specificity. Left panel—HIF1α (cyan) and ARNT (pink) DNA structure; and right panel—SIM1/ARNT2/HRE (AGGCTGCGTACGTGCGGGTCGT; flanking nucleotide contacts underlined) modelled structure (on PBD:4ZPK). Core flanking protein–DNA interactions are outlined with black arrows and red amino acids; upstream HIF1α R18 interaction with T f-4 and G f-3 (from NNCGTG).

All comparisons of DNA binding affinities were analysed and plotted using ggPlot2 in R. bHLH motifs were annotated using CAT-box (CATATG), CAG-Box (CAGCTG), CACC-Box (CACCTG), E-box (CACGTG), E-box-like [NNCGTG or DNCGTG (Fig. 1B and Supplementary Fig. S2B (ARNT2)] and random 18mer SELEX-seq affinities used to compare DNA binding specificity. In Fig. 1C comparisons of SELEX-seq DNA binding affinities of MAX (round 1) versus ARNT (round 3) was on a subset of 12-mers containing a palindromic E-box sequences (fCACGTGf) and annotated by the two nucleotides upstream of the CACGTGNN flank. Figure 1D was analysed using NRLB models to score all NNNCANNTGNNN 12-mers and annotated using the downstream nucleotide flanking the Core CANNTGf. Likewise, comparisons of bHLH-PAS TF specificity annotated and subset data to highlight differential specificity. In Fig. 2B, the 12-mer sequences predicted to be most divergent in specificity between SIM1/ARNT2 and HIF2α/ARNT [G|C]xxx[AGT]ACGTG[AA|AC|AT|CC|CT] and annotated by the NxxxxxCGTG (G = red and C = blue). All other scatterplots and boxplot comparisons are defined in the figure legends.

### ChIP-seq analysis

Bed files were used for NRLB motif model scoring in R, and motif identification and enrichment using HOMER [23] from NPAS4 ChIP-seq from mouse cortical neurons depolarized with 55 mM KCl (top 11 344 peaks) [24] (GSE21161), NPAS4, ARNT, and ARNT2 rat hippocampal neurons [8] (GSE127793), HIF1α, HIF2α, and ARNT HepG2 HK8C hypoxia-treated cells [6] (GSE120887). For HIF1α and HIF2α ChIP-seq hypoxic-treated MCF7 cells [7] (GSE28352), and AhR and ARNT ChIP-seq from TCDD-treated MCF7 cells [25] (GSE41820), reads were mapped to hg19 using Bowtie2 [26], and peaks called from sequencing data using MACS2 [27]. Intersections, manipulation of peaks, and random sequences were achieved using bedtools [28] or in R using the GRanges package [29]. Motifs from ChIP-seq peaks were identified using HOMER with the findMotifs.pl function. Receiver-operator curve analysis was implemented in R with pROC [30], comparing TF–DNA binding model-derived scores (as described in *SELEX-seq*) of ChIP-seq peaks or randomly selected sequences, and was used to calculate AUROC and associated errors. Linear regression of ChIP-seq peak scores versus motif scores was analysed and plotted as binned motif scores (0–10) versus average ChIP-seq peak scores in R. Comparison of the maximal binding score versus AUC of all scored binding events at each peak found little difference in the ability to identify NPAS4 ChIP-seq peaks [24] (AUROCmax = 0.69 versus AUROCauc = 0.67), and we used maximal score per peak for subsequent analysis. All statistical comparisons were analysed using R.

### DNAShape analysis

To investigate DNA shape contribution to the binding affinity of bHLH-PAS TFs, we initially used shapelyzer [31] to investigate TF–DNA binding model-derived shape affinity relationships. We also used DNAShapeR package to analyse Kmers aligned around the ‘Core’ CGTG using 14-mer affinity tables from fixedCore SELEX-seq to estimate shape parameters and created affinity-binned mean shape profiles to investigate shape-sequence-affinity relationships. In addition, we used NRLB TF–DNA binding models for HIF2α/ARNT or SIM1/ARNT2 to affinity-score all 10-mers downstream of a fixed upstream sequence containing the TACGTG core (HIF2α = tggAATGTGTACGTGNNNNNNNNNNcca, SIM1 = tggAAAGGGTACGTGNNNNNNNNNNcca) and shape profiles using DNAShapeR. Again, we analysed affinity-binned mean shape profiles to investigate the relationship between downstream PAS-A loop interactions with affinity-DNA shape correlations. Affinity heatmap comparisons of nucleotide co-dependencies were generated in R.

### MethylC-seq analysis

Raw FASTQ data were extracted from public repositories and 5′ read trimmed (6 bp) using fastx toolkit [19]. Trimmed data were then mapped through bismark [32] using a bisulfite-converted 1000 Genomes GRCh37 (HumanG1Kv37) reference genome. Duplicates were removed using samtools markdup [33] scheme. PileOMeth (now called MethylDackel; https://github.com/bgruening/PileOMeth) was then used to identify methylation strand bias and call CpG methylation sites based on >10× coverage per site. MCF7 datasets (three reps, two technical replicates each) are available from https://www.ncbi.nlm.nih.gov//sra/?term=SRP033283 : (i) SRR1036970 GSM1274126, (ii) SRR1036971 GSM1274127, (iii) SRR1036972 GSM1274128, (iv) SRR1036973 GSM1274129, (v) SRR1036974 GSM1274130, and (vi) SRR1036975 GSM127413. HepG2 datasets (two replicates) are available from ENCODE GSE127318 [34]. Mouse cortical, parvalbumin and vasointestinal peptide neuron data sets (two replicates) from GSE63137 [35]. Bedtools [28] was used to map methylC sites with ChIP-seq peaks and intersection of peaks performed using bedtools or Granges [29] in R.

## Structural analysis of bHLH-PAS heterodimer DNA binding

### bHLH-PAS factor homology/PAS-A loop modelling in ICM-Pro (*Molsoft ICM 3.8-6a*)

Homology models based on the mouse HIF2α:ARNT HRE DNA crystal structure (PDB: 4ZPK) were constructed in ICM-Pro [36, 37]. Peptide sequences for human ARNT (UniProt ID: P27540), ARNT2 (UniProt ID: Q9HBZ2), SIM1 (UniProt ID: P81133), and NPAS4 (UniProt ID: Q8IUM7) were prepared in FASTA format and read directly into the ICM workspace. Sequence-template alignments were made for each model to the corresponding reference chain e.g. SIM1 peptide sequence to the HIF2α protein structure. Homology models were constructed using Model Builder and subsequently refined [38]. Double-stranded DNA element models (SIM1: TGGAAAGGGTACGTGACCCGCTGCACCA; NPAS4_di: TGGAAATGGGTCGTGACCCAGGATTCCA) were built in PyMOL and refined prior to alignment by carbon-α atoms to HRE DNA. Protein/dsDNA homology models were merged and refined.

Modelling of the PAS-A loop to investigate potential DNA contacts was performed by *ab initio* interactive loop modelling of the local environment, within the internal coordinate mechanisms force field [39]. Modelling of the HIF2α:ARNT (4ZPK) PAS-A loop on HRE and SIM1 dsDNA was performed as control, as well as modelling the HRE for all homology models. A list of protein–DNA models and Oligonucleotides sequences is available in (Supplementary Table S3). Structural comparisons were made in PyMOL or Chimera and figures made using Chimera or PyMOL. Analysis of protein–DNA contacts was performed using DNAproDB [40] and manually annotated onto DNA sequences or models.

### *In silico* structure prediction and all-atom molecular dynamics simulations

As input for the all-atom molecular dynamics (MD) simulations, computational models of SIM1 (including the R171H mutant) and ARNT2 bound to SRE DNA were predicted using AlphaFold3 [41]. The tertiary and quaternary structure of each model was validated by comparing to the X-ray crystal structure of mouse HIF2α:ARNT on HRE DNA (PDB: 4ZPK) [37]. Intrinsically disordered N- and C-terminal regions were truncated for MD simulations. Each complex was prepared for MD simulations with AmberTools24 using the Amber ff19SB force field and Parmbsc1 correction [42, 43]. Models were protonated at pH 7.4 and a salinity of 150 mM using the H++ server [44]. The protonated structures were centred in a cubic box at least 1 nm from the periodic edge boundary, solvated with TIP3P waters, neutralized with Na^+^ ions, and then supplemented with Na^+^ and Cl^−^ to achieve a 150 mM NaCl concentration. Energy minimization was performed in GROMACS using the steepest descent algorithm, followed by sequential 5 ns NVT and NPT equilibration simulations at 303 K. The restrained equilibration simulations used a 2 fs timestep with a leap-frog integrator, the LINCS hydrogen bond constraint algorithm, particle mesh Ewald (PME) long-range electrostatics, a modified Berendsen thermostat (V-rescale), and Parrinello–Rahman pressure coupling [45–50]. Unrestrained production simulations were performed in triplicate for a duration of 500 ns at 303 K with a 2 fs timestep and leap-frog integrator. Frames and energies were stored every 20 ps. Analysis was performed with the MDTraj library using custom Python scripts and visualized using Matplotlib [51].

## Analysis of SIM1 R171H mutants

### Reporter assays

pGL4-Gateway-SCP1, a gift from Alexander Stark [52] (Addgene #71 510), was used to clone in six copies of the Central Midline Element (6×CME) by a novel rolling circle amplification (RCA) and cloning procedure. Briefly, using 5′ phosphorylated CME 5′ cagagccatcactgacatctgtggcacgtacaaatttcaatgtggaaggctg 3′ and rolling circle primers RCA1 (KpnI) 5′ atatggtacctctgcagccttc 3′ and RCA2 (BglII) 5′ atatagatctgctgcagagcca 3′, 10 pmol of template Oligonucleotides was cyclized and ligated using CircLigase II ssDNA Ligase (Lucigen) supplemented with 2.5 mM MnCl_2_, 1 M betaine, 1× CircLigase II buffer, and 100 U of CircLigase II enzyme in a 20 μl reaction incubated at 60°C for 1 h and 80°C for 15 min. The reactions were then purified by phenol:chloroform cleanup and used in RCA reactions. RCA reactions were performed with 20 ng of circular ssDNA, 5 μl of 10× BstPol II buffer (NEB), 1.5 μl of 10 mM dNTPs, 1 μl of T4 Gene 32 bacteriophage protein (NEB #M0300S, 5 ng/μl), 3 μl of Dimethyl sulfoxide (DMSO), 1 μl of RCA1 (60 μM) and RCA2 (60 μM), and 0.8 μl of BstII polymerase (NEB, 8 U/μl) in a 50 μl reaction. The RCA was then performed at 65°C for 90 min followed by 55°C for 120 min. The RCA reactions were then separated by agarose gel electrophoresis, and repeat lengths of interest cloned into KpnI/BglII-digested pGL4-Scp1 and sequence verified.

Expression constructs containing N-terminal class I bHLH-PAS TFs [SIM1 (1–438), SIM1.R171H (1–438)] fused to the VP64-p65-RTA (VPR) activation domain [53] were cloned into pEF IRES puro expression plasmid by isothermal assembly. N-terminal truncated ARNT (1–503)-2Myc, ARNT2(1–455)-2Myc, Gal4DBD-ARNT (1–503)-2Myc were also cloned into pEF IRES puro expression plasmids. Reporter assays were performed in 96-well white μclear plates (Griener #655094) by seeding 1 × 10^4^ HEK293T cells/well. The following day, cells were transfected with 100 ng of pG5e1b [54] or pGL4-scp1-6xCME reporter plasmid, 0.5 ng pCI-RL (Renilla) plasmid, and 25 ng of each expression plasmid or an empty vector with PEI. Forty-eight hours after transfection firefly luciferase was assayed in plate using LARII (Promega) and measured on a GloMax luminometer.

### Generation of SIM1.R171H humanized obesity-variant knock-in mice

Mouse embryonic stem cells (C57B6/Sv12) were targeted by electroporation a SIM1 targeting construct containing exon 5 of SIM1 carrying the R171H and the loxP-floxed testis-specific CRE Neo selection cassette for selection in ES cells and Cre-mediated removal upon germline transmission [55]. Targeting was performed essentially as described in [55] and confirmed by PCR using MW21 F 5′ aggggcattgcaccattacag 3′, MW21 R 5′ cttgtagccaccgcaggtgaggccagc 3′ ACN F 5′ gaattcgcccttatcggcg 3′, ACN R 5′ aagctttcgcgagctcgag 3′, R MW25 5′ aaggctttggttcttaacttcc 3′. Knock-in mice were generated by blastocyst chimera generation and backcrossed onto C57/b6 background for five generations. Sim1 R171H genotyping was achieved with MW21/MW22/MW25 multiplexed PCR primers to detect R171H allele using KAPA Mouse Genotyping Kit.

### Mouse feeding studies

Mice were bred in accordance with The University of Adelaide Laboratory Animal Services standard procedures, and experiments approved with the animal ethics committee (approval number S-2020-027). In brief, weight gain experiments were undertaken using female mice with littermate controls from Sim1^R171H/+^ × Sim1^R171H/+^ crosses. Upon weaning at 4 weeks, mice were fed *ad labitum* a high-fat diet (HFD, SF00-219 Specialty foods) and weighed weekly for 15 weeks. Repeated measures analysis of variance (ANOVA) with Bonferroni correction for multiple comparisons was used to compare differences in weight gain between WT and R171H/+ and R171H/R171H mice on high-fat diet (0–15 weeks) from 4 weeks of age using GraphPad Prism. Number of mice per genotype is noted in figure legend. Sim1^+/+^ (*n* = 8) versus Sim1^R171H/+^ (*n* = 7) total HFD food consumed per mouse during 12-h period (dark) was measured using CLAMS cages (Columbus Instruments).

### Interaction proteomics

We performed affinity purification mass spectrometry to isolate SIM1 WT or SIM1 R171H interacting proteins. To identify potential differential interactors with the SIM1.R171H region, we employed three approaches. DSP crosslinking affinity purification mass spectrometry of SIM1 WT versus SIM1.R171H using either the N-terminally truncated (Supplementary Fig. S8A) or full-length proteins (Supplementary Fig. S8B), or nuclear native (Supplementary Fig. S8C) affinity purification mass spectrometry of full length SIM1 WT or R171H (Supplementary Table S2).

### Stable cell line generation

pEF-IRESpuro-hSIM1-2×HA-3×Flag or pEF-IRESpuro-hSIM1.R171H-2xHA-3×Flag were cloned and used to generate stable cell lines by transfection with 10 μg of plasmid into ∼70% confluent 10 cm^2^ dish of HEK293T cells. Forty-eight hours following transfection, cells were selected with 1 μg/ml puromycin for 3–4 weeks until stably expressing lines were generated.

### Whole cell full length SIM1-HF X-linking tandem immunopurification proteomics

For cross-linking mass spectrometry, we established a dithiobis(succinimidyl propionate) (DSP) (Thermo Fisher #22585) in-cell cross-linking protocol. ∼1 × 10^8^ cells per condition parent HEK293T cells, HEK293T hSIM1-2×HA-3×Flag, or HEK293T-hSIM1-2×HA-3×Flag were washed with PBS, trypsinized, and resuspended in a HEPES-buffered saline (HBS: 40 mM HEPES, pH 8.05; 150 mM NaCl). A final concentration of 1 mg/ml of DSP reagent was added to the cells, and cross-linking proceeded at room temperature for 12 min gently rocking. Cross-linking was then quenched with a final concentration of 100 mM Tris, pH 8.0, and cells washed with cold PBS. Cells were then lysed with lysis buffer [LB: 40 mM HEPES, pH 8.0, 1% Triton X-100, 10 mM β-glycerophosphate, 2.5 mM MgCl2, 2 mM EDTA, 1 mM PMSF, 2× EDTA-free protease inhibitor pellets (Roche)] at 4°C for 30 min. Igepal and NaCl were then added to lysates at a final concentration of 0.85% Igepal and 150 mM NaCl, and centrifuged at 14 000 rpm for 20 min at 4°C. The resultant cross-linked supernatant was then purified by Flag M2 resin, washing with 2× lysis buffer LB, 2× IP wash buffer (20 mM HEPES, pH 8.0, 250 mM NaCl, 1 mM EDTA, 0.1% Igepal, 1 mM PMSF, 1× protease inhibitor cocktail) and 1× TBS (25 mM Tris–HCl, pH7.4, 150 mM NaCl). The protein was then eluted off the resin with 3×Flag (250 μg/ml) peptide in 1× TBS. The eluate was then incubated with HA resin (HA resin Sigma E6779) in IP wash buffer; the resin was then washed 3× with IP wash buffer and eluted with 3 × 100 μl glycine, pH 2, before equilibration of pH by the addition of 30μl of 1 M Tris–HCl, pH 9.5.

### Native nuclear tandem immunopurification proteomics

2 × 10^8^ cells stably expressing SIM1 were used per condition for immunopurification. Briefly, cells were isolated by TEN buffer (40 mM Tris, pH 8.0, 10 mM EDTA, 150 mM NaCl), washed with PBS, and cytosolic fraction isolated by resuspension of the cell pellet in 2.5× the cell pellet volume (1–2 ml) of hypotonic buffer (10 mM Hepes, pH 8.0, 1.5 mM MgCl_2_, 10 mM KCl, 0.4% Igepal, 10% ficoll, 1× protease inhibitor cocktail, 1 mM DTT), followed by incubation on ice for 5 min and clarification of nuclei by centrifugation at 14 000 rpm 15 min at 4°C. Nuclei were then lysed in 20 mM Hepes, pH 8.0, 1.5 mM MgCl_2_, 420 mM KCl, 20% glycerol, 0.2 mM EDTA, 1× protease inhibitor cocktail, 1 mM DTT for 20min on ice and clarified by centrifugation at 14 000 rpm 30 min at 4°C. The supernatant was then diluted to ∼280 mM KCl and 0.025% Igepal with IP dilution buffer (20 mM Hepes, pH 7.9, 150 mM KCl, 1 mM EDTA, 0.1% Igepal, + protease inhibitors) and incubated with Flag M2 resin overnight at 4°C rocking. The resin was then washed 4× with IP wash buffer (20mM Hepes pH 7.9, 250mM NaCl, 1mM EDTA, 0.02% Igepal, + protease inhibitors) and eluted with 250ng/μl 3xFlag peptide in IP wash buffer. The HA purification was performed as described earlier.

### N-terminal domain X-linking immunopurification proteomics

pEFIRESpuro-hSim1.3xFlag2xStrep(1–348), pEFIRESpuro-hSim1(R171H).3xFlag2xStrep(1–348), and pEFIRESpuro-hARNT2(1-455)-Myc were cloned by gibson isothermal assembly as part of this work. 3 × 15 cm 50% confluent dishes of HEK293T cells per condition were used to co-transfect pEFIRESpuro-hSim1.3xFlag2xStrep(1–348) + pEFIRESpuro-hARNT2(1-455)-Myc + pNSEN (10 μg/10 μg/5 μg = 25 μg per plate) with PEI (3:1). Sixty hours following transfection the cells were cross-linked with DSP and prepared for flag purification as described earlier. However, the lysis buffer was modified to LB2 - (20 mM HEPES, pH 8.0, 1% Triton X-100, 420 mM NaCl, 10% glycerol, 1.5 mM MgCl2, 0.2 mM EDTA, 1 × EDTA-free protease inhibitor). Flag resin was incubated with clarified lysates and washed 3 × lysis buffer LB2 and 2× IP wash buffer II (20 mM Hepes, pH 8.05, 250 mM NaCl, 0.02% Igepal, 1 mM EDTA, 5% glycerol). The cross-linked protein complexes were then eluted with IP wash buffer II supplemented with 250 ng/μl 3×Flag peptide.

All immunopurifications were run on an SDS–PAGE gel reversing disulphide crosslinks (with 50 mM DTT) and stained with either syproRuby (Thermo Fisher, #S21900) or silver stained (SilverQuest, Thermo Fisher LC6070). Proteomics was performed by FASP trypsin digest and cleanup, tryptic peptides were identified by Nano-LC − ESI-MS/MS an LTQ Orbitrap XL ETD MS instrument (Thermo Fisher Scientific) or QTOF mass spectrometer (Bruker Daltonics) at the Adelaide Proteomics Centre. For the 3×Flag peptide eluted samples these were thoroughly washed on FASP vivacom 30 kDa molecular weight cutoff filters prior to trypsinization to remove 3×Flag peptide contamination.

### Mass spectrometry analysis

Peptide identification and label-free quantification of mass spectrometry data were performed using MAXquant [56] with a mass error tolerance of 20 ppm, and Perseus [57] was used for normalization, filtering, and imputation. For comparison of interacting proteins between SIM1 WT and SIM1.R171H, we subtracted mock purifications from immunopurified samples, log transformed the data, cluster normalized in Perseus and Pearson’s correlation was calculated on the label-free quantification of each protein for SIM1 WT versus SIM1.R171H. To analyse relative coimmunopurification of ARNT or ARNT2 with SIM1 WT or SIM1 R171H, we combined label free quantification of ARNT and ARNT2 peptides. Interacting proteins for all proteomics experiments can be found in Supplementary Table S4.

### Co-immunopreciptation analysis

Co-immunoprecipitation of interacting proteins identified by interaction proteomics was performed essentially as described in [15] using Flag M2 (A2220) or HA7 (E6779) agarose resins (Sigma). pEF-IRESpuro-hSim1-2myc and pEF-IRESpuro-hSim1.R171H-2myc were described previously [58], pEF1a-Ronin-FLAG-IRES-Neo was a gift from Thomas Zwaka (Addgene plasmid # 28020) [59], pcDNA Myc DBC1 was a gift from Osamu Hiraike (Addgene plasmid # 35 096) [60], pKH3-TRIM28 was a gift from Fanxiu Zhu (Addgene plasmid # 45 569) [61], and pCGN-HCF-1 fl was a gift from Winship Herr (Addgene plasmid # 53 309) [62].

### CRISPR knock-in of tags to endogenous HIF1α and HIF2α

CRISPR sgRNA constructs targeting adjacent to the endogenous HIF1α and HIF2α stop codons were cloned into px330 by ligating annealed and phosphorylated Oligonucleotides with BbsI digested px330, using hHIF1α sgRNA upper 5′ caccgTGAAGAATTACTCAGAGCTT 3′, hHIF1α sgRNA lower 5′ aaacAAGCTCTGAGTAATTCTTCAc 3′ or hHIF2α CTD sgRNA upper 5′ caccgCCTCCTCAGAGCCCTGGACC 3′, hHIF2α CTD sgRNA lower 5′ aaacGGTCCAGGGCTCTGAGGAGGc 3′. Knock-in of HA-3xFlag epitopes into the endogenous HIF1α or HIF2α locus in HepG2 cells was achieved by transfection with 0.625 μg of pNSEN, 0.625 μg of pEFIRES-puro6, 2.5 μg of px330-sgHIFa CTD, and 1.25 μg of ssDNA HDR template Oligonucleotides containing flanking homology to CRISPR targeting site the tag insertion and a PAM mutant into ∼0.8 × 10^6^ cells using polyethylenimine (PEI, 3:1). Forty-eight hours after transfection, the medium was removed from cells and replaced with fresh medium supplemented with 2 μg/ml puromycin for 48 h and then cell medium was changed to fresh medium without puromycin. Forty-eight hours later cells were seeded by limiting dilution into 96-well plates such that an average of 0.5 cells/well were present. Correct integration of the tags into the endogenous loci was identified by PCR screening using HIF1α gDNA screen F 5′ ggcaatcaatggatgaaagtggatt 3′, HIF1α gDNA screen R 5′ gctactgcaatgcaatggtttaaat 3′, and HIF2α gDNA screen F 5′ taccaacccttctttcaggcatggc 3′, HIF2α gDNA screen R 5′ gcttggtgacctgggcaagtctgc 3′ and positive colonies reisolated as single colonies by limiting dilution. Isolated HIF1α and HIF2α tag insertions were confirmed by PCR, sanger sequencing and western blotting.

HepG2 cells were grown in normoxia or <1% oxygen and 5% CO_2_ using a hypoxia workstation for 4 or 16 h. Cells were then washed with PBS prior to lysis with whole cell extract buffer (20 mM Hepes, pH 8.0, 420 mM NaCl_2_, 0.5% Igepal, 0.2 mM EDTA, 1.5 mM MgCl_2_, 25% glycerol) supplemented with 2 mM DTT and 1× protease inhibitors. Extracts were run on 7.5% SDS–PAGE gels and transferred to nitrocellulose prior to western blot detection with anti-HA (HIFa, HA11–16B12; MMS-101R), anti-β-tubulin (Biorad) and species specific HRP conjugated secondary antibodies (Peirce).

### Statistical information

Statistical comparisons were implemented using R or Prism and indicated in the main text or figure legends.

### Data and code availability

SELEX sequencing data for fixed core and random 18mer data sets are available on the Gene Expression Omnibus (GEO) as GSE159989. Kmer Affinity tables are also available at GSE159989. NRLB models and ProBound models presented here and used for scoring sequences are provided in the Supplementary materials.

## Results

### DNA binding preferences for the bHLH-PAS TF family

Chromatin selectivity and target gene selection are guided by multiple mechanisms including TF DNA binding specificity and stoichiometry, chromatin accessibility, nucleosome positioning, histone interactions, TF cooperativity, and DNA and histone modifications [63–66]. We reasoned that bHLH-PAS TF target gene selectivity is driven by multiple mechanisms, including factors affecting chromatin selectivity in addition to inherent DNA binding specificity. As such we sought to collectively understand some of the underlying mechanisms of bHLH-PAS TF chromatin selectivity.

Initially, to define the DNA binding specificity of the bHLH-PAS TF family, we sought to profile hierarchies of DNA sequences bound by the most distinctive members of the family (Supplementary Fig. S1A). Class I bHLH-PAS TFs prototypically form heterodimer pairs with either ARNT or ARNT2. To facilitate profiling of the DNA response elements of the bHLH-PAS TF family, we purified full-length (ARNT or ARNT2) or N-terminal truncated (Class I bHLH-PAS) dimers from mammalian Expi293 cells (ARNT or ARNT2; full-length), baculovirus-infected Sf9 insect cells (HIF1α/ARNT, HIF2α/ARNT, SIM1/ARNT2, NPAS4/ARNT2; bHLH-PASA + B domains), or bacteria (AhR/ARNT; bHLH-PASA domains) (Supplementary Fig. S1B). Dimer pairs were selected based on predicted diversity of DNA binding response elements and the most probable endogenous complexes [5]. For example, ubiquitously expressed HIF1α and AhR have been shown to act in heterodimeric complexes with ARNT, whereas NPAS4 and SIM1 have been shown to partner ARNT2 in neuronal cells. ARNT and ARNT2 have also been shown to form homodimers, which we also analysed, despite the *in vivo* significance of these homodimers remaining unknown.

Initially, *in vitro* DNA binding preferences of several demarcated bHLH-PASA-PASB purified dimers (ARNT/ARNT, ARNT2/ARNT2, AhR/ARNT, HIF1α/ARNT, HIF2α/ARNT, SIM1/ARNT2, and NPAS4/ARNT2) were determined using SELEX sequencing (SELEX-seq) [18, 20] (Supplementary Fig. S1A and B). The SELEX libraries contained either a random 18mer library or libraries containing a fixed E-Box-like (CGTG) core flanked by 8 random nucleotides upstream and 10 nucleotides downstream (Fig. 1A), in an attempt to capture the full specificity upstream and downstream of the core-response element (NNCGTG; core nucleotides 1–6 = c1–c6). By comparing sequence-affinity relationships for all TF complexes using the two approaches of Kmer affinity tables [20] and NRLB energy models [21], high concordance in affinities was found in multiple rounds of selection and in the relative affinities and TF selectivity from different library strategies (Supplementary Fig. S2A–J).

We generated affinity tables for 10mers (random 18mer library) or 12mers (18/22mer fixed core) and compared the selection of the nucleotide directly upstream of the Core CGTG (i.e NCGTG). We found strong concordance between round 1 or round 2 affinities generated for fixed core 12mers affinities (Supplementary Fig. S2A; HIF2α/ARNT r1 v r2 *r*^2^* = *0.85, SIM1/ARNT2 r1 v r2 *r*^2^* = *0.92, AhR/ARNT r1 v r2 *r*^2^* = *0.86, NPAS4/ARNT2 r1 v r2 *r*^2^ = 0.92, Pearson’s correlation coefficient). In addition, we also observed that both library selection strategies strongly enriched for TF-specific NCGTG response elements (Supplementary Fig. S2B–D). Consistent with previous reports, we found that ARNT, ARNT2, HIF1α, HIF2α, and SIM1 selectively bind ACGTG containing-elements, whereas NPAS4 (TCGTG) and AhR (GCGTG) bind distinct core elements (Supplementary Fig. S2C and D). As expected, ACGTG-binding bHLH-PAS TFs can be further distinguished by the nucleotide upstream of the ACGTG (NACGTG). ARNT and ARNT2 highest affinity sites select for CACGTG-containing response elements, whereas HIF1α/ARNT, HIF2α/ARNT and SIM1/ARNT2 complexes select for TACGTG-containing response elements, NPAS4/ARNT2 select for GTCGTG-containing response elements, and AhR/ARNT select for TGCGTG-containing response elements (Fig. 1F and Supplementary Fig. S2C and D). These data show that this *in vitro* system accurately recapitulates core response element preferences of bHLH PAS TFs that have previously been described *in vitro* and *in vivo*.

### Flank encoded binding specificity between bHLH TFs

While members of the bHLH superfamily of TFs can bind to various core (CAT-box, CACC-box, CAG-Box, E-box (CACGTG), or E-box-like (DNCGTG)) sequences [1, 67], we found that ARNT homodimers bound selectively to the E-Box palindrome (CACGTG) and bHLH-PAS heterodimers bound selectively to E-Box like sequences (DNCGTG; D = A,T,G) (Fig. 1B and Supplementary Fig. S2B). Given that E-Box elements are also bound by other non-PAS bHLH TFs such as MAX and MyoD, and that DNA-binding specificity of yeast bHLH TFs can be distinguished by the nucleotides flanking the core E-Box [68], the DNA binding affinities of MAX [21] versus ARNT were compared. First, protein–DNA binding models from SELEX-seq data were generated and energy logos representing DNA binding affinities of MAX or ARNT across a 12mer footprint were created (Fig. 1C). Indeed, comparision of CACGTG-containing 10mers or modelled affinities indicated that core flanking nucleotide preferences (CACGTGff; f_+1_,f_+2_) were different between ARNT and MAX and suggested selective recruitment of different bHLH subfamily TFs to distinct extended E-Box elements (Fig. 1C and D).

Within the bHLH-PAS heterodimers tested, selective DNA binding between different complexes was encoded by a single nucleotide at the 5′ of the core (NNCGTG or NNCGTG; c_1_ or c_2_) (Fig. 1E and Supplementary Figs S2C–J and S3A–F). An exception was observed for the HIFα isoforms, where binding affinities were indistinguishable (HIF1α/ARNT versus HIF2α/ARNT; *r*^2^ = 0.94; Supplementary Fig. S2H). Similar to HIFα, poorly selective DNA binding was observed between the closely related homodimers of ARNT versus ARNT2 (*r*^2^ = 0.77; Supplementary Fig. S2G). We also noted that while AhR/ARNT and NPAS4/ARNT2 exhibit distinct, high affinity core DNA binding motifs, those for SIM1/ARNT2, HIF1α/ARNT, and HIF2α/ARNT appear to be indistinguishable (Fig. 1F and Supplementary Fig. S2I and J and S3H and I). However, NRLB energy logos (Fig. 1F) indicate that additional specificity encoded in flanking sequences may be sufficient to mediate selectivity between otherwise identical TF binding sites. A recent biophysical approach to model DNA binding affinities known as ProBound has recently been developed, whilst similar in its approach to NRLB, ProBound incorporates additional parameters, integrates selection rounds and employs a different computational approach [22]. To determine whether the NRLB models were recapitulated using an orthogonal method, we modelled SELEX data using ProBound. We found that models were near indistinguishable from the NRLB and were also highly predictive of SELEX read counts (Supplementary Fig. S3H and I, *r*^2^ = 0.99, *P* = 0.97). This indicates that both NRLB and ProBound models were highly accurate representations of the *in vitro* binding of the bHLH-PAS TFs.

### ARNT versus ARNT2 and HIF1α versus HIF2α isoform specificity is not explained by differential affinity for DNA motifs

ARNT and ARNT2 have been shown to have some distinct biological functions despite significant overlapping expression patterns in neurons and other tissues [5]. We generated NRLB models for ARNT and ARNT2 (Supplementary Fig. S2G) and compared 10mer affinities for ARNT and ARNT2 DNA binding and found a high degree of correlation between affinities (Supplementary Fig. S2G; Pearson’s *r*^2^ = 0.77). Although ARNT appears to be highly restricted to CACGTG-containing response elements, ARNT2 may be able to bind to a more flexible consensus motif (NACGTG or CGCGTG, albeit with much lower affinity than the core CACGTG) (Supplementary Fig. S2C).

HIF1α and HIF2α display overlapping and distinct expression patterns as well as biological functions in the adaptation to low oxygen [69]. These disparate functions are proposed to be mediated by the distinct HIF1α− or HIF2α-dependent target gene regulation, for example HIF1α regulates metabolic genes whereas HIF2α regulates genes involved in erythropoiesis and iron homeostasis [69]. HIF1α and HIF2α also show distinct genomic occupancy within the same cell types, with HIF1α binding more prevalently to promoter regions whereas HIF2α binds more prevalently to enhancers [6, 7, 70, 71]. Low resolution analysis of DNA binding specificity from chromatin immunoprecipitation sequencing experiments (ChIP-seq) [6] [RCGTG (R = A or G)] has not sufficiently addressed whether inherently different HIF1α and HIF2α DNA binding specificities might explain genomic site selection preferences. Therefore, we compared the DNA binding specificity of HIF1α/ARNT and HIF2α/ARNT 10mer affinity tables (Supplementary Fig. S2I and J) and found a near identical DNA binding specificity (*R^2^*= 0.94). As expected, both HIF1α and HIF2α bound strongly to ACGTG-containing cores, less efficiently to GCGTG-containing cores, and most efficiently binding to TACGTG (Supplementary Fig. S2D and I–J). We also found that HIF1α/ARNT and HIF2α/ARNT dimers bound with similar affinity (HIF1α ∼2 fold higher affinity than HIF2α) to TACGTG-containing probes in EMSA analyses, consistent with previous reports [37]. This indicates that the HIF1α/ARNT and HIF2α/ARNT *in vitro* specificity and affinity is near identical and is therefore unlikely to contribute to HIFα genome binding selection (Supplementary Fig. S4A–C). Given that there are both overlapping and distinct HIF1α and HIF2α chromatin binding sites, we hypothesized that sequential loading or competition for HIF binding sites may be influenced by the hypoxic induction dynamics or stoichiometry of HIF1α or HIF2α. Therefore, we generated C-terminally HA-3xFlag (HF)-tagged heterozygous HIF1α or HIF2α knock-in HepG2 cells using CRISPR/Cas9-mediated homologous directed repair (Supplementary Fig. S4D). We did not observe isoform-specific differences in the dynamics of hypoxic induction at < 1% O_2_, with both HIF1α and HIF2α displaying hypoxic protein induction at 4 and 16 h in HepG2 lines (2 × HIF1α.HF lines and 1 × HIF2α.HF line) (Supplementary Fig. S4E). However, we observed large differences in the relative protein stoichiometry with much greater HIF1α levels than HIF2α in HepG2 cells (Supplementary Fig. S4E). This suggests that sequential loading of chromatin by HIF1α and HIF2α is not a mechanism for chromatin site selection and that HIF1α is unable to occupy HIF2α enhancer sites, even when in significant excess to HIF2α. This is also supported by recent evidence showing that HIF1α and HIF2α are unable to occupy each other’s preferential binding sites even if the other isoform is removed [6].

### Extended motif DNA-binding models predict ChIP-seq chromatin occupancy and affinity

Next, we generated energy logos from the NRLB/ProBound models for all SELEX-seq data to determine specificity outside of the core motif (Supplementary Figs S3G and H and S5B). NRLB models derived from either round 1 or 2 or integrated ProBound models of the FixedCore library approach were similar and revealed extensive flanking nucleotide preferences (Fig. 1E and Supplementary Fig. S3G and H). Consistent with previous validation of the accuracy of NRLB modelling approach [21] we confirmed the ability of models to accurately predict DNA binding.

Firstly, we validated flanking nucleotide contribution to binding affinity by EMSA of NPAS4/ARNT2 heterodimers on the top 22mer sequence derived from NRLB models compared to flanking variants of this sequence (Supplementary Fig. S5A). We found that variants upstream or downstream of the invariant core (GTCGTG) contributed to binding affinity (Supplementary Fig. S5A). We found a linear relationship between predicted binding affinity and comparative EMSA probe binding (Supplementary Fig. S5B; *r*^2 ^= 0.94), indicating that models were able to predict flanking nucleotide contribution to binding affinity. Next, we compared the ability of bHLH-PAS DNA binding models to predict ChIP-seq peaks [6–8, 24, 25] by energy model-based scoring of ChIP-seq peaks as compared to random genomic regions using area under receiver operator curves (AUROC) to assess model performance.

We found that NPAS4/ARNT2 TF–DNA binding models accurately predict NPAS4 occupancy at NPAS4, NPAS4/ARNT, or NPAS4/ARNT2 peaks, and performed better than ARNT or ARNT2 models (Supplementary Fig. S5C; rat PTX AUROC = 0.69–0.72). We also found that models were better at discriminating strongly bound peaks versus weakly bound peaks (Supplementary Fig. S5C and D, rat PTX; AUROC = 0.68 versus 0.92, mouse KCl; AUROC 0.63 v 0.79). We reasoned that DNA binding affinity would result in increased ChIP-seq peak intensity. As such, we also compared the model affinity scores to *in vivo* ‘affinity’ using linear regression by comparing mean ChIP-seq peak intensity versus binned [1–10] predicted motif affinity. We found a highly significant relationship between NPAS4 affinity scores and NPAS4 ChIP-seq peak intensity (mouse and rat *P* < 2 × 10^−16^, Supplementary Fig. S5D and E).

We also compared the ability of ARNT, HIF1α/ARNT, or HIF2α/ARNT (random 18mer and 18/22 FixedCore) derived models to accurately discriminate ChIP-seq peaks in HepG2 cells. While all models were able to accurately identify ChIP-seq peaks, HIFα/ARNT models performed best at predicting HIFα/ARNT shared peaks (AUROC > 0.9 at HIFα/ARNT peaks) (Supplementary Fig. S5F). HIF1α and HIF2α MCF7 ChIP-seq comparisons also confirmed model prediction of ChIP-seq peak intensity (Supplementary Fig. S5F–H; HIF1α ChIP-seq , HIFα model AUROC = 0.75, HIF2α ChIP-seq , HIFα model AUROC = 0.68). Taken together, this validates the ability of *in vitro* generated TF models to accurately predict and quantify both *in vitro* and *in vivo* binding.

### Core-flanking DNA-encoded interactions mediate SIM1 versus HIFa DNA binding specificity

Several of the bHLH-PAS TFs have been previously described to bind identical motif’s, including heterodimeric (ARNT or ARNT2) forms of SIM1, SIM2, HIF1α, HIF2α all binding to TACGTG sequences. Indeed, SIM1/ARNT2, HIF1α/ARNT and HIF2α/ARNT bound to near identical core binding sequences (Supplementary Fig. S2C and D). However, energy logos indicated that sequence specificity outside of the core may further contribute to selectivity.

To examine this, we initially compared kmer affinities for each TF dimer on the high affinity IUPAC consensus sequences, confirming highly specific DNA binding for NPAS4 and AhR on their preferred consensus sites (Supplementary Fig. S6A). In addition, it also suggested that SIM1 and HIF2α can bind preferentially to their individual consensus sequences (Supplementary Fig. S6A). We also showed that SIM1/ARNT2 preferentially bound to its top predicted 22mer DNA probe as compared to the top predicted 22mer DNA probe for HIF2α/ARNT, indicating that SIM1/ARNT2 favours distinct flanking sequences to HIF2α/ARNT (Supplementary Fig. S6B).

Comparing energy logos from all HIFα/ARNT (HIF1α and HIF2α) and SIM1/ARNT2 SELEX-seq experiments demonstrated that different upstream nucleotide preferences exist between HIFα/ARNT and SIM1/ARNT2, with TG nucleotide preference for HIFα/ARNT at f_-3_-f_-4_ upstream of the core sequence (NNNNTACGTG) (Fig. 2A). In addition, comparison of HIF2α/ARNT and SIM1/ARNT2 fixed core energy logos indicated that both upstream and downstream nucleotides likely contribute to specificity (Supplementary Fig. S6A–E). This was supported by comparison of kmer (subsequence) affinities of preferential upstream and downstream sequences, demonstrating HIF2α and SIM1 preferential binding to identical core (TACGTG) response elements *in vitro (*Fig. 2B and Supplementary Fig. S6B–E). The *in vitro* specificity of HIFα/ARNT heterodimers was also reflected by increased occupancy of HIFα at GNNTACGTG-containing HIF1α ChIP-seq peaks (and less pronounced for HIF2α) in both HepG2 [6] and MCF7 cells [7], supporting *in vitro* DNA binding preferences being consistent with *in vivo* chromatin site selection (Fig. 2C). In addition, linear regression coefficients of HIFα model-based scoring of HIFα ChIP-seq peaks were higher than SIM1 models and larger than other bHLH-PAS models, indicating that the more intense HIFα/ARNT ChIP-seq peaks are encoded by flanking DNA specificity *in vivo* (Fig. 2D). Taken together, these observations indicate that HIFα/ARNT flanking nucleotide specificity can direct HIFα *in vivo* DNA occupancy and provide evidence for how target gene specificity between closely related members of the bHLH-PAS family is achieved.

To further investigate mechanisms that direct specificity differences between HIFα/ARNT heterodimers versus SIM1/ARNT2 heterodimers, the structures of HIF2α/ARNT in complex with a hypoxia response element (HRE) [37] (PDB: 4ZPK) and modelled structure of SIM1/ARNT2/HRE were compared (Fig. 2E and G). Analysis of protein–DNA interactions revealed extensive contacts upstream and downstream of the core DNA binding sequence (Fig. 2E and F). This included HIF2α Arg18 DNA contacts upstream (f_-3_-f_-4_ of the core binding site, NNNNTACGTG) at the site of preferential HIF2α DNA binding, which results from an N-terminal extension of the basic DNA binding domain that is absent in SIM1 (Fig. 2F and G). This indicates that HIFα may have evolved unique specificity to overcome DNA site competition with other bHLH-PAS TF complexes that preferentially bind a TACGTG core.

### AT-Hook domains in bHLH-PAS TFs control DNA binding *in vivo* and underlie Sim1 R171H obesity etiology

While no DNA contacts were identified that could explain the proximal (f_+1_/f_+2_ TACGTGNN) downstream specificity between HIF2α/ARNT and SIM1/ARNT2, the distal (f_+5_-f_+8_) downstream nucleotide preferences revealed a preference for AT-rich sequences close to PAS loop contacts (Fig. 2G). We hypothesized that distal AT-rich specificity may be indicative of more complex shape requirements to accommodate high-affinity DNA binding. To examine this, k-mer affinity tables and energy models were used to investigate nucleotide co-dependencies and DNA shape parameters such as Minor Groove Width (MGW), Propeller Twist (ProT), Helical twist (HelT), or Roll [72].

**Figure 3.**
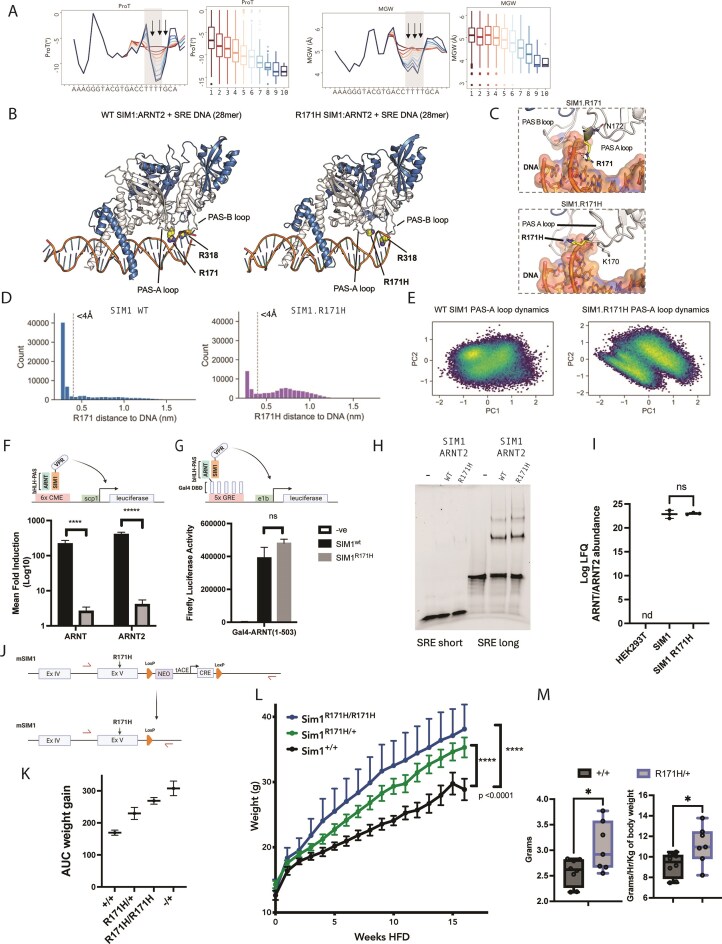
PAS-A interactions distal to the core binding sites are associated with AT-rich, narrowed MGW and reduced ProT revealing SIM1.R171H as a driver of monogenic hyperphagic obesity. (**A**) SIM1/ARNT2 DNA shape analysis of mean ProT and MGW affinity binned (low = red, blue = high, bins = 0–10) by TF–DNA binding model scored downstream 22mers. ProT and MGW boxplots at position at T17 (middle arrow; f_+7_). (**B**) Representative snapshots of WT (left) and R171H (right) SIM1/ARNT/SRE AlphaFold3 models from the MD simulations (SIM1—grey, ARNT2—blue) show SIM1 PAS-A loop extension into the major groove at nucleotides T16 and T17 (correlating with the positions for shape distortion arrows in panel (A). (**C**) Hydrogen bonding of R171 but not R171H to the phosphate backbone of DNA. (**D, E**) MD simulations of SIM1/ARNT2/SRE (left) or SIM1.R171H/ARNT2/SRE (right). (**D**) Histogram of the minimum distance (in Angstroms) of the Arg guanidinium or His imidazole atoms to DNA from each frame of the replicate 500 ns MD trajectories. (**E**) Principle component analysis of the cartesian coordinates of the PAS-A loop. (**F, G**) SIM1(1-348)-VPR or SIM1.R171H(1-348)-VPR were coexpressed with (**F**) ARNT(1-503) or ARNT2(1–455) and a 6×TACGTG luciferase reporter, (**G**) Gal4-ARNT(1-503) and a 5× GRE reporter to assess TF activity and dimerization, respectively. (**F**) Mean fold induction ARNT or ARNT2 alone was calculated for each replicate and then the log_10_ transformed prior to statistical tests. (**F, G**) Statistics were calculated comparing the multiple unpaired t-tests. **** *P* = .000626, *****P = .000013. *n* = 3 (± SEM) independent experiments (**H**) EMSA of purified hSIM1(1-348)/hARNT2 (69-438) or hSIM1.R171H(1-348)/hARNT2 using 22mer SRE probes or SRE 79mer. hSIM/hARNT2 DNA binding is weaker on shorter versus longer probes but unchanged comparing WT and R171H, *n* = 3, EMSAs. (**I**) Immunopurification (mock (HEK293T) versus SIM1 WT versus SIM.R171H) proteomics (see methods and Supplementary for details) and label free quantification was used to assess the ability of SIM1 WT or SIM1.R171H to dimerize with ARNT or ARNT2. Mean (± 95% CI). nd = not detectable, ns = not significant *P* = 0.7661 unpaired two tailed t-test. (**J**) SIM1.R171H knock-in mouse model construct and generation. Primer sites are indicated with red arrows. (**K**) mean (±SEM (+ for SIM^R171H/R171H^ for clarity) weight gain of SIM1 WT (*n* = 8), versus SIM^R171H/+^ (*n* = 12) versus SIM^R171H/R171H^ (*n* = 5) littermates over a period of 16 weeks on a high fat diet (HFD). Inset is the AUC over 16 weeks weight gain on a HFD for SIM1 WT, SIM1^R171H/+^, SIM1^R171H/R171H^, or SIM1^−/+^ (*n* = 3). Sim1^+/+^ versus Sim1^R171H/+^  *P* = 0.0000007568, Sim1^+/+^ versus Sim1^R171H/R171H^  *P* = 0.0000002663, ****P < 0.0001. (**M**) (left panel) Average of the total HFD food consumed per mouse during 12 h period (Dark). (Right panel)—Hourly food consumption rate for each mouse, averaged over the 12 h dark (active) period and corrected for bodyweight. SIM1 WT versus SIM1 Mut (SIM1^R171H/+^) showed increased average food consumption (2.573 ± 0.2625 versus 3.067 ± 0.4813, *n* = 8, *P* = 0.0257) and increased food consumption rate (9.210 ± 1.163 versus 11.17 ± 1.824, *n* = 7, *P *= 0.0258). Data mean ± sd and analysed by unpaired two-tailed *t*-test.

**Figure 4.**
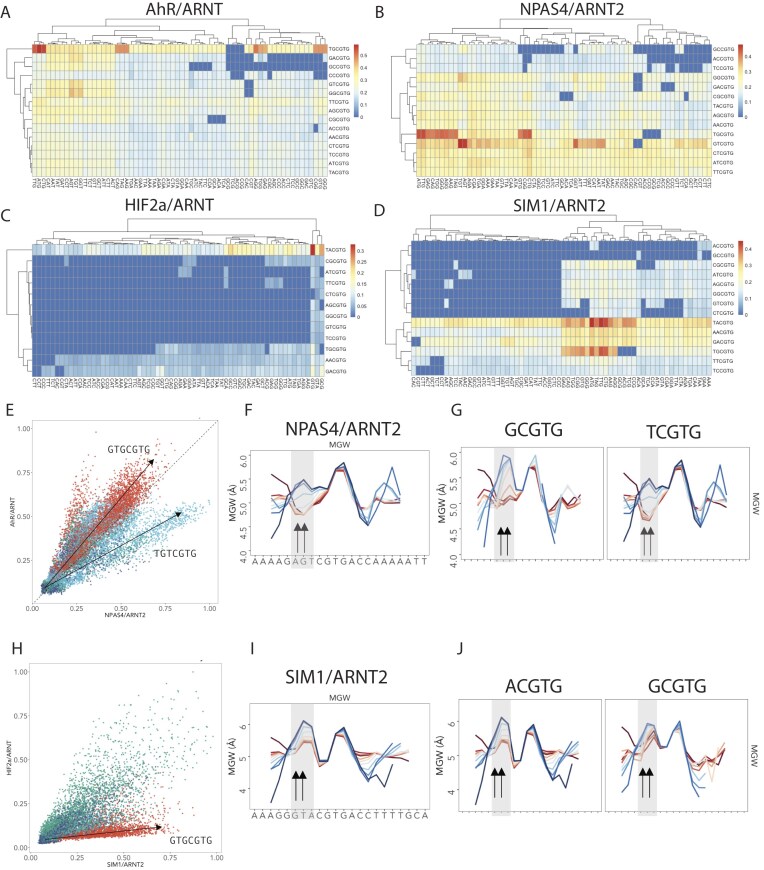
Upstream nucleotide co-dependencies encoded by DNA shape confer TF selectivity. (**A**–**D**) 12mer Affinities (oneCGTG per line) comparing Core NNCGTG (y) and upstream trinucleotides NNNxxCGTG (x). Heatmaps comparing NNNxxCGTG versus NNCGTG for (**A**) AhR/ARNT or (**B**) NPAS4/ARNT2. (**C**) HIF2α/ARNT or (**D**) SIM1/ARNT2. (**E**) Scatterplot comparison of k-mer affinities AhR/ARNT (y) versus NPAS4/ARNT2 (x) subset for and coloured by GTG(1–3)CGTG (red—GTGCGTG, light blue = GTGNCGTG, green = GTGNNCGTG, dark blue = GTGNNNCGTG). (**F, G**) increased minor groove width (MGW) at NNNCGTG correlates with increased affinity (high = blue, low = red) for (**F**) NPAS4/ARNT2 all k-mers, (**G**) NPAS4/ARNT2 k-mers subset up [T/G]CGTG). (**H**) Scatterplot comparison of k-mer affinities HIF2α/ARNT (y) versus SIM1/ARNT2 (x) subset for and coloured by GTG(1–3)CGTG (red—GTGCGTG, lightblue = GTGNCGTG, green = GTGNNCGTG, darkblue = GTGNNNCGTG). (**I**-**J**) increased minor groove width (MGW) at NNNCGTG correlates with increased affinity (high = blue, low = red) for (**I**) SIM1/ARNT2 all k-mers SIM1/ARNT2 k-mers subset up [A/G]CGTG).

High affinity AT-rich downstream sequences (f_+5_-f_+8_) were associated with decreased ProT, and MGW associated with high-affinity binding in both SIM1/ARNT2 and HIF2α/ARNT (Fig. 3A and B and Fig. 4I and J). While structural analysis of HIF1α/ARNT/HRE and SIM1/ARNT2/HRE models indicate a distal interaction of a PAS-A loop with DNA (Fig. 2 F and G), several characteristics of the structures [37] and SELEX-seq data led us to hypothesize that this may not be the optimal DNA-bound conformation. Firstly, nucleotide enrichment extended beyond the proposed PAS-A loop-DNA contact and the likely flexible PAS-A loop contains classic DNA binding residues (K and R), which may allow the loop to reorient into a more favorable (lower energy binding conformation). In addition, AT-rich regions are often bound by chromatin-associated proteins containing AT-hook domains [73, 74]. Intriguingly, alignment of HIF1α and HIF2α PAS-A loops (pal) with AT-Hook motifs from MeCP2 and HMGB1 revealed a conserved RGR AT-hook interaction motif (Supplementary Fig. S7A and B).

We also noticed that the combination of shorter DNA response elements and crystal contacts between DNA of symmetry mates observed in structures may constrain the structural conformation of heterodimer on DNA, which may not reflect the optimal in-solution conformation in which the SELEX-seq was performed.

Given these observations we generated homology models with energy minimization for SIM1/ARNT2, HIF2α/ARNT, NPAS4/ARNT and NPAS4/ARNT2 on longer consensus DNA sequences derived from SELEX-seq DNA motifs and performed PAS-A loop modelling to better capture the distal DNA contacts (Fig. 3B and C and Supplementary Fig. S7C and D). Structural loop remodelling enabled PAS-A loop extension more distally, allowing Arg/Lys residues in the loop to reorient to come in close proximity or interact with DNA at a conserved position for HIF2α, SIM1 and NPAS4 (Fig. 3B and C and Supplementary Fig. S7C and D), indicating distal DNA sites may be common to bHLH-PAS TFs. Specifically, Arg 181 in HIF2α and Arg 171 in SIM1 extend into the major groove at the site of AT-enrichment (Supplementary Fig. S7C and D) whereas R158 in NPAS4 models adopt different loop conformations with ARNT or ARNT2 indicating structural flexibility. This may allow surveying of the downstream DNA and possibly explain differences in downstream TF–DNA binding model nucleotide enrichment between HIF2α/SIM1 and NPAS4. While others [37, 75, 76] have indicated that the PAS domains, either PAS-A or PAS-B [76], make DNA or histone contacts, the biological importance of these interactions remains unclear.

Mutations in the AT-hook domain of MeCP2 have been shown to lead to Rett syndrome intellectual disability in humans and mouse models through reduced nucleosome DNA interaction [77–79], indicating that mutations in other AT-Hook domain containing proteins may be important for disease etiology. Indeed, we found several variants in clinical databases or from previous early onset obesity cohorts which are predicted to disrupt PAS-loop-mediated DNA binding in NPAS4, HIF2α, or SIM1 (Supplementary Fig. S7E).

Sim1 is critical for hypothalamic satiety signaling and loss-of-function leads to hyperphagic obesity in mice. Loss of SIM1 activity is also associated with early onset obesity and Prader-Willi like features in humans [80–82]. One human SIM1 early onset obesity variant, SIM1.R171H [80], is within the PAS-A -AT-Hook region predicted to contact DNA (Supplementary Fig. S7A) and has been shown to possess only 30% of wildtype (WT) SIM1 activity, a trait that is shared with its paralog SIM2 [58, 80]. To understand the effect of the SIM1.R171H variant on contact with DNA we further generated AlphaFold3 models [41] of SIM1/ARNT2 or SIM1.R171H/ARNT2 on longer consensus DNA binding sequences derived from high affinity TF–DNA binding model motifs and performed all-atom MD simulations to probe the interaction of the R171 and R171H with DNA (Fig. 3B and C). This confirmed the close proximity of the R171 to DNA just beyond the AT-rich SELEX sequences and that the R171H was directed away from DNA (Fig. 3B and C). It also indicated that PAS-B reorientates to come in close proximity to DNA but beyond structural model probes or SELEX variable regions (Fig. 3B and C). Simulations of the R171 and R171H interaction with DNA demonstrate that for the bulk of each of the trajectories, the R171 is within <4 Å from DNA (contacting) whereas the R171H is >4 Å away, and indicating that R171 interacts with the DNA phosphate backbone and nucleotide bases (Fig. 3C and D). Furthermore, principle component analysis of the PAS-A loop indicates substantially different geometry and dynamics of the PAS-A loop with R171 or R171H. Together this indicates that R171 and (Fig. 3C) additionally PAS-B R318 may contact DNA contributing to DNA binding strength or altering the conformation of the protein which may be important for chromatin interactions or transactivation.

Multiple mouse models have shown that haploinsufficiency in Sim1 results in hyperphagic obesity [82–85], however whether partial loss-of-function is sufficient to drive obesity is also yet to be determined. This is of particular interest as the majority of SIM1 variants that were found to be associated with obesity resulted in modest partial loss of function (30%–80% of WT) [80, 81]. As such, whether these are sufficient to drive monogenic obesity or are polygenic in nature is an important unexplored question. In addition, the biological contribution of PAS-A loop-DNA interactions described here or PAS-A-DNA or PAS-B-histone interactions described by others [37, 75, 76] has not been addressed. Therefore, we explored the importance of distal AT-hook interactions to obesity etiology by investigating SIM1.R171H loss of function.

To further define SIM1.R171H mechanism we utilized reporter gene assays designed to individually assess DNA/Chromatin binding and dimerization (Fig. 3F and G), and interaction proteomics (Fig. 4H and Supplementary Fig. S8A–E). Using a SIM1-responsive reporter construct with the DNA binding region (bHLH-PAS-A-B) of SIM1 fused to a VPR activation domain, in conjunction with ARNT or ARNT2 DNA binding regions (bHLH-PAS-A-B) lacking the transactivation domains, we found that SIM1 R171H failed to activate the reporter, indicating SIM1 R171H has a lower affinity or transactivation activity (Fig. 3F). In contrast, SIM1-VPR and SIM1.R171H-VPR (bHLH-PAS-A-B) expressed with Gal4-ARNT (bHLH-PAS-A-B) activated a Gal4 responsive reporter equally (Fig. 3G), indicating that loss of function is a result of altered DNA or chromatin binding and not dimerization with ARNT. To determine whether DNA binding strength was altered in SIM1.R171H mutants or there was an involvement of PAS-B, as suggested by AlphaFold3 models (Fig. 3B and C), in inherent DNA binding we compared *in vitro* DNA binding of SIM1/ARNT2 versus SIM1.R171H/ARNT2 on SRE containing 22mers or 79mers (with SELEX probe constant regions). AlphaFold3 models of SIM1/ARNT2 (Fig. 3B and C) suggested that PAS-B may also contact DNA beyond 22mers, probes as such comparing long and short probes may indicate whether PAS-B is important for DNA binding. Indeed, we found dramatic increase in DNA binding with longer probes but no difference in the ability of SIM1.R171H to bind DNA *in vitro* (Fig. 3H).

To further exclude confounding effects of loss of dimerization and/or altered protein-protein interactions as an explanation for SIM1.R171H loss of function we performed affinity purification mass spectrometry to isolate SIM1 WT or SIM1 R171H interacting proteins (Supplementary Fig. 8A–E). In order to identify potential differential interactors with the SIM1.R171H region we employed three approaches: DSP crosslinking affinity purification mass spectrometry of SIM1 WT versus SIM1.R171H using either the N-terminally truncated (Supplementary Fig. 8A) or full-length proteins, (Supplementary Fig. 8B) or nuclear native (Supplementary Fig. 8C) affinity purification mass spectrometry of full length SIM1 WT versus SIM1.R171H (Supplementary Table 2). All affinity purification methods isolated SIM1 as the most abundant protein and ARNT and ARNT2 as known interacting proteins. In addition, as expected the predominantly cytoplasmic HSP90A was identified in whole cell extract purifications but lowly abundant in nuclear extract purifications. We also identified many TF or chromatin binding proteins consistent with the function of SIM1 as a TF.

We found that ∼97% of N-terminal interacting proteins identified as interactors of SIM1 WT were also found as SIM1.R171H interactors. In addition, label free quantification of interactors comparing all experiments demonstrated that SIM1 WT and SIM1.R171H displayed no difference in the ability to pull-down ARNT/ARNT2 (Fig. 3I). Quantification of SIM1 WT vs R171H interacting proteins was highly consistent within each purification strategy (Pearson’s *r*^2^ = 0.84–0.99), and we did not identify any significantly different interactors between SIM1 WT and SIM1.R171H when comparing all interaction proteomics datasets. In addition, we confirmed that there was not differential interaction of proteins between SIM1 WT and SIM1 R171H by selecting proteins that displayed differential interaction in a single experiment and performing co-IP experiments of these selected interactors (Supplementary Fig. 8E). We found that none of the interacting proteins identified by mass spectrometry preferentially interacted with SIM1 WT. Taken together, we conclude that SIM1.R171H loss of function is a result of altered DNA and or chromatin binding and not altered dimerization with ARNT, ARNT2, or other interacting proteins, consistent with the PAS-A loop role in DNA binding.

In order to investigate the biological consequence of PAS-A loop DNA binding mutant R171H and its possible contribution to hyperphagia induced obesity we generated a knock-in mouse model for SIM1.R171H (Fig. 3J). We found that SIM1^R171H/+^ or SIM1^R171H/R171H^ mice gained significantly (*P* < 10^−6^, repeated measures ANOVA) more weight on a high-fat diet over a 15-week period as compared to littermate WT controls with an associated increased food consumption (Fig. 3K–M). This indicates that partial loss of SIM1 function in heterozygotes is sufficient to drive hyperphagia-induced obesity as a result of distal DNA interactions made by the SIM1 PAS-A loop.

### Multi-modal DNA binding underlies specificity within the bHLH-PAS family

In addition to downstream shape-mediated DNA binding we also noted that nucleotides upstream of the core were associated with shape-influenced affinity relationships in bHLH-PAS TFs. By comparing the nucleotide co-dependencies in k-mer affinities upstream (NNNNNCGTG; f_-1_-f_-3_) of the core (Fig. 4A–D) strong upstream co-dependencies in NPAS4/ARNT2 and SIM1/ARNT2 DNA binding sequences were observed that contribute to TF specificity (Fig. 4E and H). In addition, AhR/ARNT and NPAS4/ARNT2 bound with similar affinity to GTGCGTG sequences, which appears to be shape encoded by increased MGW upstream of the core (CGTG), allowing NPAS4/ARNT2 a more flexible DNA binding motif (GTGCGTG or GTGTCGTG) as compared to AhR/ARNT (GTGCGTG) (Fig. 3E) and providing mechanistic detail to previous observations that NPAS4 was able to bind both TCGTG and GCGTG with high affinity [10]. NPAS4/ARNT2 appears to bind with the same affinity to GTGCGTG and GTGTCGTG which have similar MGW–affinity profiles, indicating that NPAS4/ARNT2 may bind DNA through a shape-directed mechanism (Fig. 4F and G). Similarly, SIM1/ARNT2 heterodimers bind core sequences via a shape-encoded mechanism that enables specificity between TF dimers. SIM1/ARNT2 bound strongly to GTACGTG and GTGCGTG sequences, through an associated increased MGW, whereas HIF2α/ARNT weakly bound GTGCGTG, contributing to additional specificity between SIM1/ARNT2 and HIF2α/ARNT DNA binding (Fig. 4H–J). This indicates that multiple core binding specificity for SIM1 and NPAS4 is also an important undescribed mechanism to encode DNA binding specificity between bHLH-PAS family members.

### DNA methylation appears to mediate cell type-specific DNA binding by bHLH-PAS TFs

**Figure 5.**
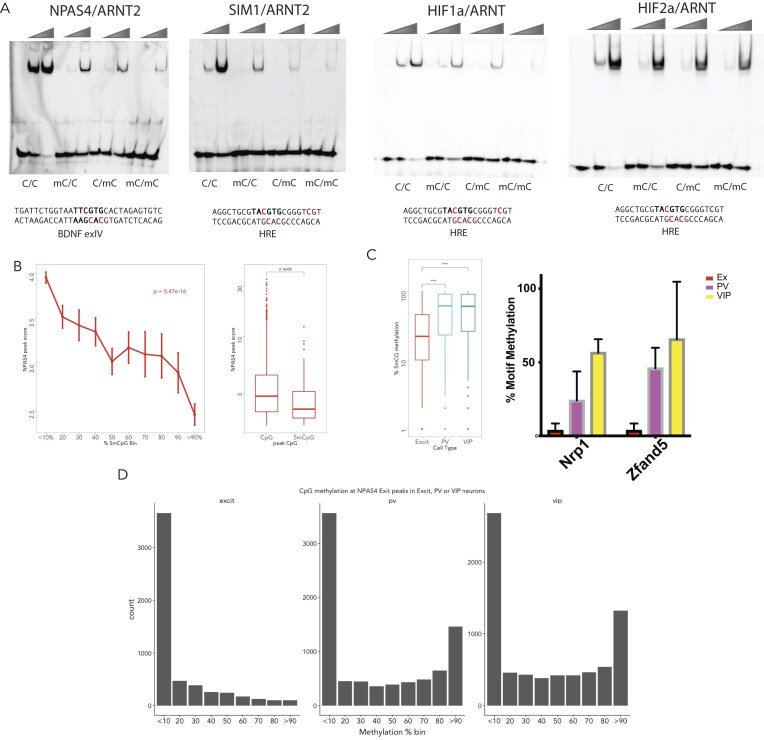
bHLH-PAS TFs are sensitive to response element CpG methylation, which controls cell type specific TF occupancy. (**A**) NPAS4/ARNT2 on the BDNF IV promoter NPAS4 response element (TTCGTG), SIM1/ARNT2, HIF1α/ARNT, and HIF2α/ARNT on the hypoxia response element (TACGTG). Increasing amounts of purified protein was incubated with the indicated probes, which were unmethylated (C/C), methylated on the top strand (mC/C), the bottom strand (C/mC), or both strands (mC/mC) as indicated in the sequence below the EMSA gel (methylated = red). (*n* = 3). (**B**) (left panel) linear regression of comparing binned % CpG methylated DNA at NPAS4 occupied TF sites from depolarized mouse neurons peaks to average ChIP-seq peak score. (model *P*-value is inset). (right panel) mean log_10_ NPAS4 ChIP-seq peaks score at unmethylated (CpG, <10%) or methylated (mCpG, >90%) sites. (**C**) (left panel) Average CpG methylation at NPAS4 ChIP-seq peaks (CTX, excitatory neurons, 11 344) in excitatory cortical neurons , inhibitory parvalbumin (PV) neurons , or inhibitory vasointestinal peptide (VIP) neurons. Wilcoxon statistical test **** *P* < 10^−200^. (right panel) Excitatory specific NPAS4 regulated genes Nrp1 and Zfand5 are methylated at NPAS4 ChIP-seq peaks in inhibitory neurons. Average CpG methylation (*n* = 2) at NPAS4 ChIP-seq peaks (CTX, excitatory neurons) in excitatory cortical neurons, inhibitory parvalbumin (PV) neurons, or inhibitory vasointestinal peptide (VIP) neurons. (**D**) Number NPAS4 ChIP-seq peaks (CTX, excitatory neurons) of peaks in each % CpG methylation bin (0%–100%) for excitatory cortical neurons (left panel), inhibitory parvalbumin (pv) neurons (middle panel), or inhibitory vasointestinal peptide (vip) neurons (right panel).

Interfamily and intrafamily DNA binding specificity and target gene selectivity appears to be mediated by sequence encoded mechanisms. However, HIF1α and HIF2α display identical DNA binding preferences, yet they have unique chromatin occupancies within the same cell types [6, 7] and unique target genes. In addition, NPAS4 has been shown to have opposing roles on synapse function and gene regulation in inhibitory versus excitatory neurons [86]. We hypothesized that DNA response element methylation may play a role in directing bHLH-PAS TF occupancy *in vivo*. We performed gel shifts using response elements methylated at various CpG and CpH positions within the core response element and downstream PAS interaction site for bHLH-PAS heterodimers NPAS4/ARNT2, SIM1/ARNT2, HIF1α/ARNT, and HIF2α/ARNT (Fig. 5A). We found that in all cases CpG and/or CpH methylation was able to reduce the affinity of bHLH-PAS TFs for their cognate response elements with partial blocking of DNA binding when only the Watson strand or the Crick strand was methylated (Fig. 5A).

To investigate whether Cp methylation directs TF occupancy *in vivo*, we analysed NPAS4 ChIP-seq data demonstrating a negative linear relationship between CpG methylation at ChIP-seq peaks and NPAS4 ChIP-seq peak intensity as well as a significantly higher NPAS4 peak score (*P* < 10^−9^) at unmethylated peaks versus methylated peaks and a large proportion of highly methylated (>90%) sites in inhibitory neurons (parvalbumin and vasointestinal peptide) (Fig. 5B and D). In addition, mean CpG methylation at excitatory NPAS4 ChIP-seq peaks was significantly higher (*P* < 10^−16^) in inhibitory neurons compared to excitatory neurons (Fig. 5C). Highly methylated TF binding sites were also observed at excitatory neuron -specific NPAS4 target genes (Nrp1 and Zfand5) in inhibitory neurons, suggesting that DNA methylation may direct NPAS4 target gene selection (Fig. 5C). To investigate whether this is a common mechanism for cell-selective chromatin occupancy for the bHLH-PAS TFs, we then compared HIF1α and HIF2α ChIP-seq data sets in MCF7, HepG2, HKC8, and RCC4 cells with DNA methylation profiles from HepG2 or MCF7 cells. Consistently, we observed significantly higher CpG methylation at cell type specific HIFα ChIP-seq peaks compared to shared peaks (Supplementary Fig. S9A–H). In addition, we also observed significantly lower methylation at HIF1α and HIF2α shared peaks as compared to unique peaks, but no significant difference in methylation between HIF1α and HIF2α unique peaks (Supplementary Fig. S9I and J). We found that while HIFα occupied sites appeared to be predominantly unmethylated, we could not identify differential methylation of HIF2α specific sites, indicating that other mechanisms are likely at play. Overall, DNA methylation does appear to have a significant role in cell type-specific DNA occupancy by bHLH PAS TFs.

## Discussion

Here we define the *in vitro* DNA binding specificity for the bHLH-PAS TF family. We find that, contrary to previous reports, the specificity of the bHLH-PAS heterodimers extends far beyond the previously identified 6–10mer sequences. We find that bHLH-PAS heterodimers bind 12–15bp motifs with substantial upstream and downstream specificity. We demonstrate that *in vitro* motifs can classify chromatin-bound TFs and contribute to chromatin affinity. Furthermore, structural analysis of heterodimer-DNA complexes mechanistically supports a role for SELEX-identified sequence specificity in core flanking regions. For example, N-terminal flank specificity differences between HIF/ARNT and SIM1/ARNT can be explained by N-terminal HIFa DNA contacts which are absent for SIM1, an observation which is further supported by chromatin sequence preferences for HIF1α. This important finding of undiscovered specificity mediated by domains or regions outside of the prototypical DNA binding domain outlines the need to revisit the inherent specificity of other families of TFs with seemingly overlapping specificity. This is especially pertinent to families of TFs where near identical DNA binding specificity is observed in *in vitro* DNA binding experiments, yet *in vivo* chromatin occupancy and function are somewhat non-overlapping [64].

We also find specificity that extends beyond the core is both sequence- and shape-encoded which allows promiscuity in DNA binding. Integration of DNA bound structural and TF DNA binding models indicates that with PAS-A and PAS-B loops may be involved in contacting DNA downstream of the core DNA binding site. Shape-encoded TF–DNA specificity for SIM1/ARNT2 and HIF2α/ARNT downstream of the core also revealed a novel AT-Hook-like domain within the PAS-A-loop of HIF1α and HIF2α, with similarity to HMGA1 and MeCP2 AT-Hook domain amino acid sequences (Supplementary Fig. S7B). Alignment of the PAS loop domain (Supplementary Fig. S7A) of other class I bHLH-PAS TFs indicates that basic residues within the loop may mediate an interaction with DNA distal of the core DNA binding site as indicated by SELEX-seq and energy logo enrichment. Indeed, mutations in other residues within the loop have previously been shown to lead to reduced DNA binding [37], transactivation [58, 80, 81] or target gene activation (see Supplementary Fig. S7A) [87]. We also observe several other variants in HIFα and NPAS4 in the key Arg residues in the PAS-A loop from clinically relevant cohorts [88] that are likely to perturb DNA interaction, providing a mechanism for observed clinical outcomes (Supplementary Fig. S7E). Importantly, while we and others observe biochemical PAS-A or PAS-B interactions with chromatin or DNA, here we demonstrate that this is indeed biologically important for *in vivo* function using the SIM1.R171H mouse model. We show that the R171 residue which lies within the AT-hook like PAS-A loop contacts the phosphate DNA backbone at the site of downstream SELEX sequence specificity. Furthermore, the human hyperphagia-associated early onset obesity mutation SIM1.R171H results in reduced reporter activation without defects in ARNT dimerization or cofactor recruitment, indicating deficits in DNA binding. Using a knock-in mouse model carrying the human obesity variant SIM1.R171H we demonstrate that PAS-A loop contacts are important for biological function and disease as both SIM1^R171H/+^ and SIM1^R171H/R171H^ gain substantially more weight on a high fat diet.

The PAS domains have been proposed to contact DNA outside the Core NNCGTG motif to convey additional DNA specificity [12] or strength [16, 37, 89]. Our *in vitro* evidence supports the PAS-A loop regions directly contacting DNA through a sequence/shape directed mechanism, which is supported by *in vivo* loss of function in the Sim1.R171H mice leading to hyperphagic obesity (Fig. 3J–M). The additional observation that PAS-B may also contact DNA combined with strong DNA binding on longer probes indicate that PAS-A and PAS-B interactions may both be required for DNA binding or chromatin specificity *in vivo*. Although *in vitro* DNA binding of SIM1 WT versus R171H remained unchanged, we observed much reduced transactivation on reporters suggesting that R171 may interact with chromatin or is required for a DNA mediated conformational change that promotes transactivation. Indeed, PAS loop mediated interactions for CLOCK/BMAL have recently been described and mutation of relevant residues only modestly affects chromatin and not naked DNA binding *in vitro* [76]. In addition, we have also described a Y323H PAS-B Loop mutation that leads to reduced transcriptional activity in SIM1, indicating that the PAS-B loop may also contact DNA or chromatin to increase transcriptional activity. Taken together this indicates that PAS-A and B loops in bHLH-PAS proteins may play multiple roles in *in vivo* DNA/Chromatin binding specificity, strength and/or conformation and ultimately transactivation, although this is yet to be explored.

Beyond the 3′ distal interactions and TF–DNA binding specificity we also show that SIM1/ARNT2 and NPAS4/ARNT2 can bind with similar affinity to multiple distinct core DNA sequences. Multiple TF–DNA binding specificity has also been described for several distinct TFs in particular FOXN3 [90], HOXB13 and CDX2[91]. The mechanisms that underlie bispecific DNA binding of NPAS4/ARNT2 and SIM1/ARNT2 as well as the influence this has on chromatin specificity and transcriptional activation is yet to be explored. In addition, whether this TF–DNA binding flexibility is conferred to ARNT containing complexes or restricted to SIM1/ARNT2 and NPAS4/ARNT2 remains unclear.

Intriguingly, the presence of AT-rich selection downstream of the core sequence NNCGTG, appears to match a preference for MeCP2 binding at AT-rich sequences downstream of CmG sites [73, 79, 92]. In addition, analysis of CpH methylation sites in neurons reveals a propensity of CAC (GTG) CpH sites [93] which also appear to have a downstream preference for A [94] and are also preferentially recognized by MeCP2 [73]. This represents an attractive mechanism by which MeCP2 may specifically target methylated bHLH-PAS motifs. Indeed, we find that bHLH-PAS TF dimers are sensitive to response element methylation which appears to contribute to cell-type specific chromatin occupancy (Fig. 5 and Supplementary Fig. S9).

In summary, we describe several important and unique features of motif recognition and DNA-protein interaction that explain interfamily specificity (bHLH (MAX) versus bHLH-PAS), intrafamily specificity through core bHLH-PAS motif differences, and flanking sequences that contribute to core shared intrafamily specificity. We show that bHLH-PAS TFs bind DNA through a shape-directed mechanism that can dictate both core promiscuity and specificity as well as downstream flanking preference. We also identify novel distal PAS-A loop interactions with downstream DNA sites that are important for DNA binding, protein conformation or chromatin interactions, offering explanation for the underlying cause of human hyperphagic obesity by the SIM1 R171H variant, which we recapitulated in a SIM1.R171H mouse model, and predict similar mechanisms for clinically relevant NPAS4 and HIF-2α variants.

## Supplementary Material

gkaf1352_Supplemental_Files

## Data Availability

SELEX sequencing data for fixed-core and random 18-mer data sets are available on the Gene Expression Omnibus (GEO, https://www.ncbi.nlm.nih.gov/geo/) as GSE159989. K-mer affinity tables are also available at GSE159989. Code used here was implemented predominantly in R. Code and NRLB models used for scoring bed files are available at https://github.com/BeeSting-pgm/TF_BED_Score and https://zenodo.org/doi/10.5281/zenodo.13340507.
