# Supplementary material for: Multimodal bHLH-PAS DNA binding controls specificity and drives obesity
Source: Nucleic Acids Res. 2026 Jan 6;54(1):gkaf1352. doi: 10.1093/nar/gkaf1352 (PMC12774657; doi:10.1093/nar/gkaf1352)
Supplement: gkaf1352_Supplemental_Files [file gkaf1352_supplemental_files.zip › Multimodal bHLH-PAS DNA binding controls specificity and drives obesity_SupplementaryFigures_280325_FINAL_B.pdf]

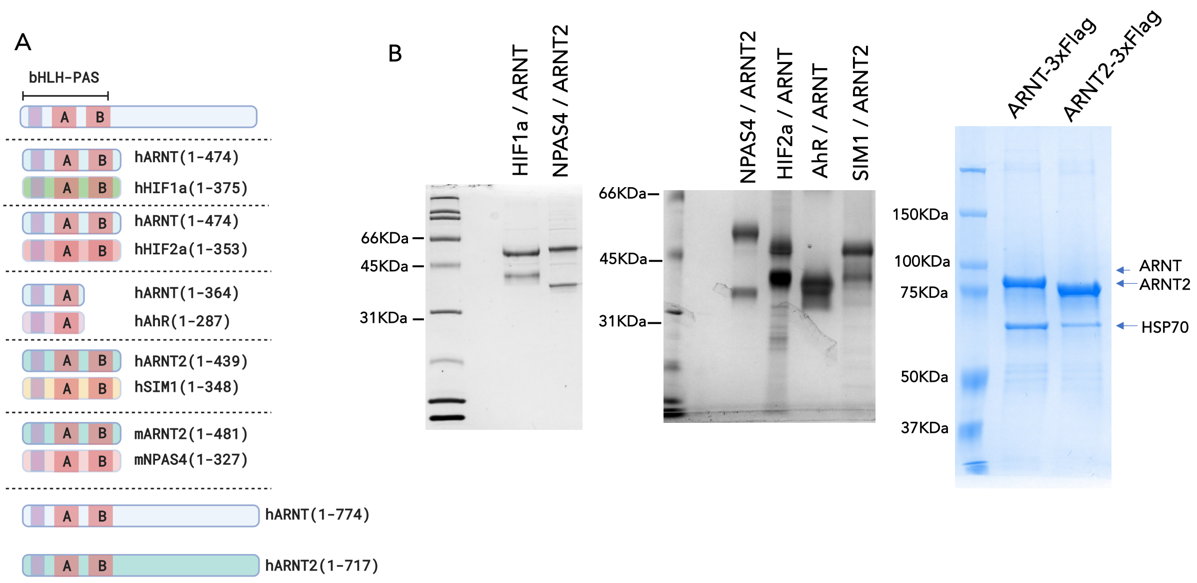

Supplementary Figure 1. Transcription Factor truncations and proteins used in SELEX-seq. A.) Schematic diagram of the different bHLH-PAS transcription factor constructs used in SELEX-seq and *in vitro* DNA binding analyses. **B.)** SDS-PAGE gel analysis of purified dimeric bHLH-PAS proteins used in this study, *note AhR and ARNT are co-migrating and HSP70 contaminant in ARNT purifications.

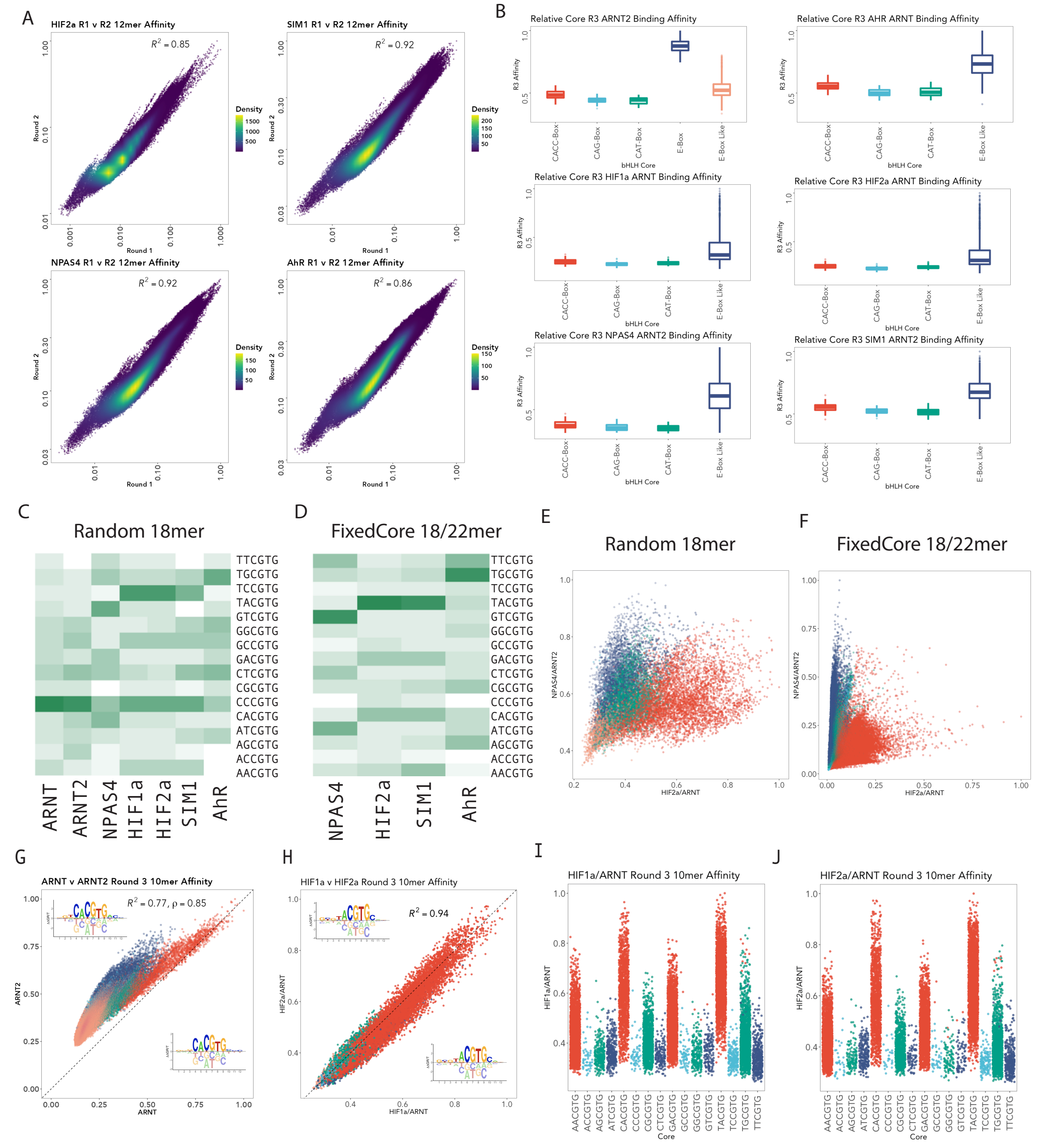

Supplementary Figure 2. **Comparison SELEX-seq analysis strategies and Core DNA binding specificity of the bHLH-PAS transcription factors. A.)** Comparison of the relative affinity of 12mer affinities from Round 1 or Round 2 of FixedCore 18/22mer SELEX-seq data filtered for one CGTG per sequence. Log$_{10}$ 12mer affinities generated from SELEX-seq analysis were plotted comparing Round 1 (x-axis) and Round 2 (y-axis) for HIF2α/ARNT, SIM1/ARNT2, AhR/ARNT or NPAS4/ARNT2. r^2= pearsons correlation coefficient. **B.)** Round 3 Kmer Affinity boxplots for each transcription factor on different bHLH motifs. **C.)** and **D.)** Heatmaps comparing relative 10mer affinities for the labeled bHLH-PAS transcription factor complexes on each core NNCGTG DNA binding site generated using **C.)** Random 18mer library SELEX strategy (Round 3) or **D.)** the FixedCore 18/22mer SELEX strategy (Round 2). **E.)** and **F.)** Differential bHLH-PAS DNA binding specificity is encoded by distinct core NCGTG sequences. Comparison of relative affinities of NPAS4/ARNT2 vs HIF2α/ARNT using **E.)** 10mers (Random 18mer library SELEX strategy (Round 3)) or **F.)** 12mers (FixedCore 18/22mer SELEX strategy (Round 2)). **G-H.)** Comparison of Relative 10mers Kmer Affinities (Random 18mer library SELEX strategy (Round 3)) (Inset 12mer NRLB models) **G.)** ARNT vs ARNT2, r^2 = 0.77, p = 0.85 **H.)** HIF1α/ARNT vs HIF2α/ARNT2, r^2 = 0.94. Relative Core NNCGTG 10mer Affinities of each probe for **I)** HIF1α/ARNT or **J.)** HIF2α/ARNT (Random 18mer library SELEX strategy (Round 3)). In all scatterplot figures (Red = ACGTG, Dark Blue = TCGTG, Green = GCGTG, light Blue = CCGTG, pink = No Core).

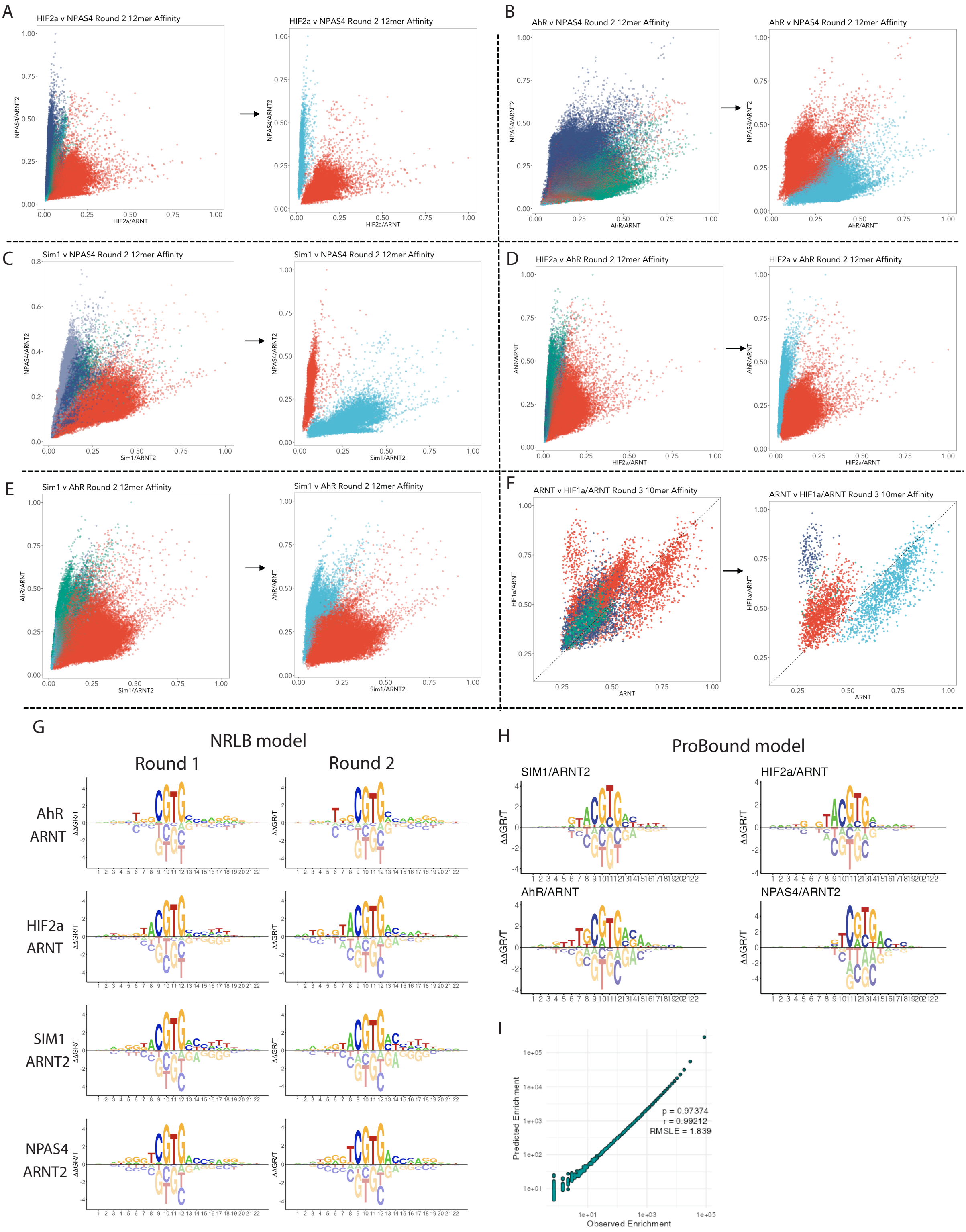

Supplementary Figure 3. Core encoded specificity and energy models of the bHLH-PAS transcription factor family. A-F.) Kmer Affinity scatter plots Core specificity colour by NCGTG (left panel) or the most divergent NNCGTG right panel. **A.)** NPAS4/ARNT2 v HIF2α/ARNT (Round 2 12mers - fixedCore 18/22mer) left panel (ACGTG - red, TCGTG – darkblue), right panel (GTCGTG - blue, TACGTG - red). **B.)** NPAS4/ARNT2 v AhR/ARNT (Round 2 12mers - fixedCore 18/22mer) left panel (GCGTG - darkblue, TCGTG – green), right panel (GTCGTG - red, TGCGTG - blue). **C.)** SIM1/ARNT2 v NPAS4/ARNT2 (Round 2 12mers - fixedCore 18/22mer) left panel (TCGTG - red, ACGTG – mauve), right panel (GTCGTG - red, TACGTG - blue). **D.)** HIF2α/ARNT v AhR/ARNT (Round 2 12mers - fixedCore 18/22mer) left panel (TCGTG - green, ACGTG – red), right panel (TACGTG - blue, TGCGTG - red). **E.)** SIM1/ARNT2 v AhR/ARNT (Round 2 12mers - fixedCore 18/22mer) left panel (GCGTG - green, ACGTG – red), right panel (TGCGTG - blue, TACGTG - red). **F.)** ARNT/ARNT v HIF1α/ARNT (Round 3 10mers - random 18mers) left panel (GCGTG - darkblue, TCGTG – green, ACGTG – red), right panel (CACGTG - lightblue, TACGTG – darkblue, GACGTG - red). Energy Logos generated from FixedCore **G.)** NRLB or **H.)** ProBound models. NRLB non-symmetrical models were generated by modeling on SELEX-seq filtered data (one central CGTG per read) from Round 1 (left panel) or Round 2 (right panel) for AhR/ARNT, HIF2α/ARNT**,** SIM1/ARNT2 and NPAS4/ARNT2. **I)** Validation of ProBound Model SIM1/ARNT2 prediction of observed probe enrichments from selex data. Data is observed vs the predicted enrichment of binned probes, spearman's Rho (p) and Pearsons (r) correlations between observed and predicted as well as the regression Root Mean Squared Logarithmic Error (RSMLE) are displayed.

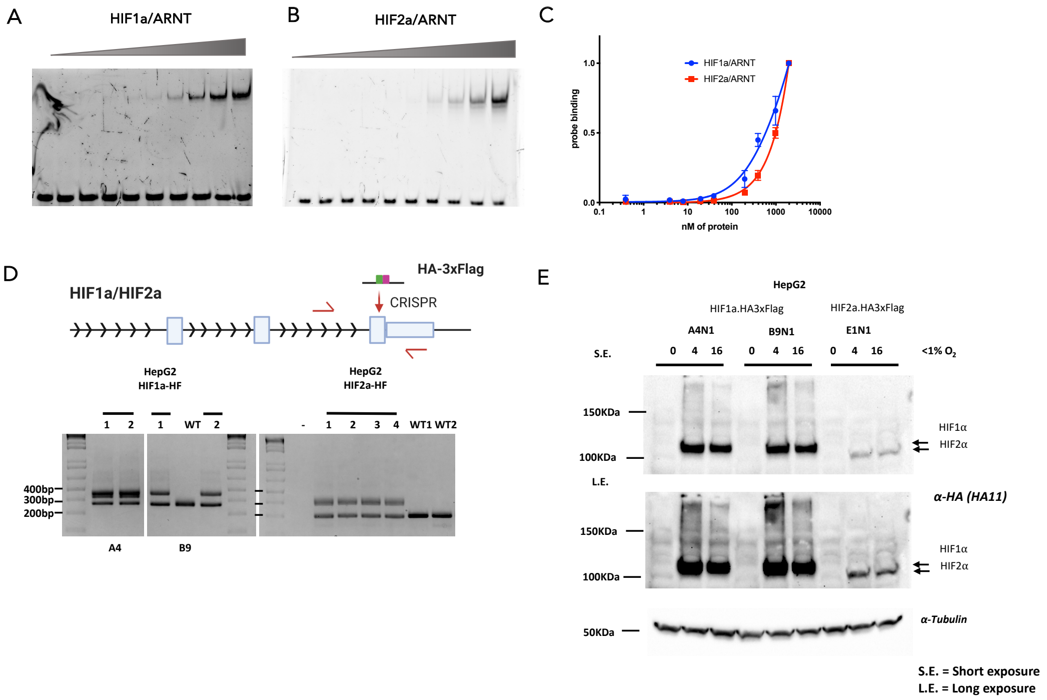

Supplementary Figure 4. Characteristics of HIF1α vs HIF2α DNA-binding, dimerization and stoichiometry. **A.)** and **B.)** DNA binding Affinity of HIF1α/ARNT and HIF2α/ARNT was assessed by EMSA. Increasing amounts of **A.)** HIF1α/ARNT or B.) HIF2α/ARNT heterodimer were incubated with a FAM labeled HRE probe. **C.)** Relative HRE probe binding from gel shift experiments (n = 3) shows similar DNA binding affinity for HIF2α/ARNT (red) or HIF1α/ARNT (blue). **D.)** Schematic diagram (upper panel) of CRISPR homology directed repair (HDR) strategy to knock-in HA-3xFlag tag into endogenous HIF1a or HIF2α locus. CRISPR guide sgRNA was designed to cut near the endogenous stop codon of HIF1α or HIF2α in HepG2 cells, and an oligo template containing flanking homology to HIF1α or HIF2α and a single HA tag and 3xFlag tags was provided as a HDR template. Cell monoclone's were isolated by two rounds of limiting dilution and genomic PCR screening for HA-Flag insertion (Using primers flanking the insertion site (red)), followed by sanger sequencing to confirm tag insertion. **E.)** Western blot of HIF1α.HAFlag and HIF2α.HAFlag tagged HepG2 knock-in monoclonal cell lines. HepG2 cells were incubated in 0.5-1% O2 chamber for 4 or 16 hrs prior to protein extraction SDS-PAGE gel electrophoresis and western blotting with anti-HA or anti-Tubulin antibodies.

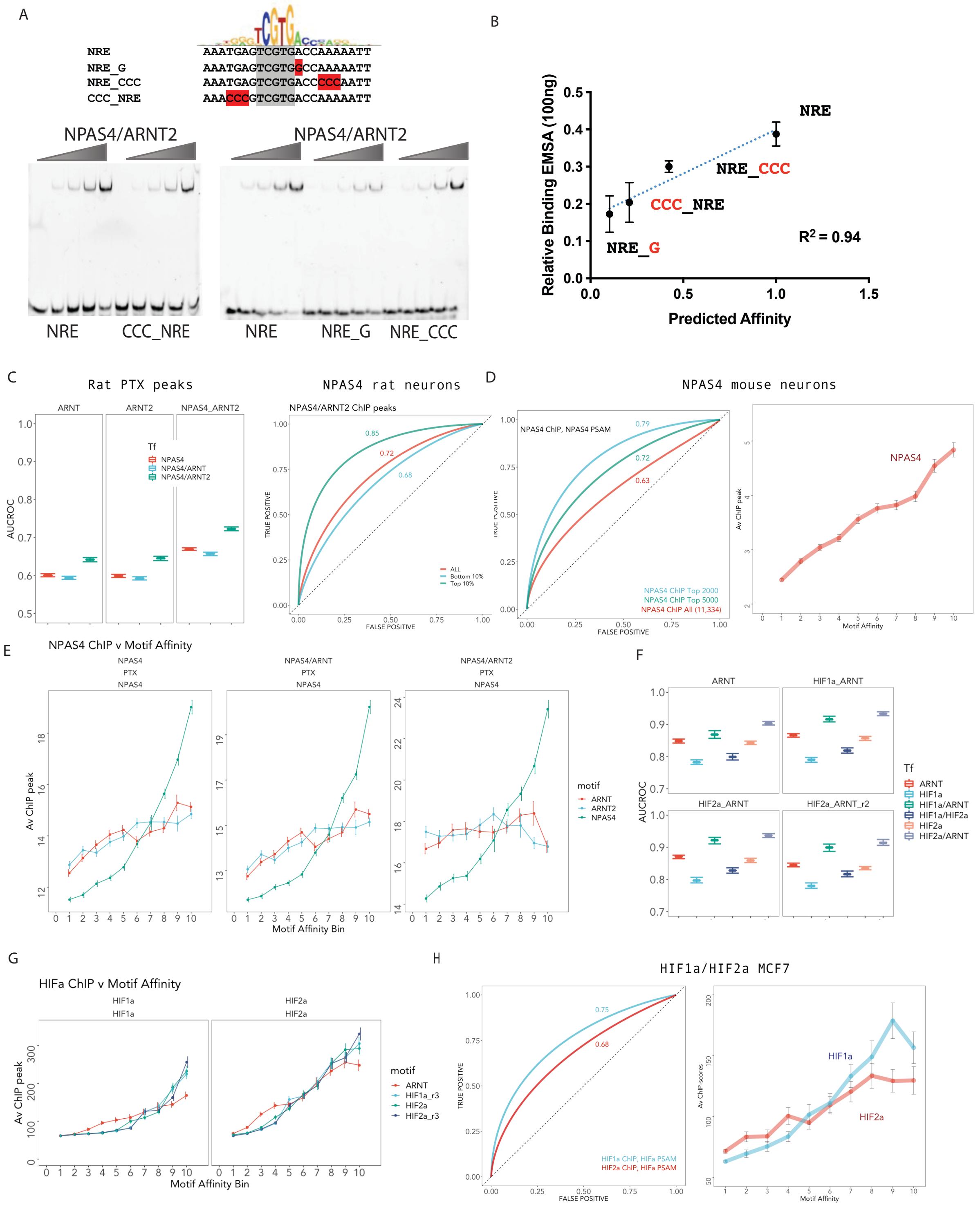

Supplementary Figure 5. Validation of DNA binging Energy Models.

A.) NRLB Energy Logo and EMSA probe sequences used to compare flanking nucleotide contribution to DNA binding. EMSA of increasing amounts of NPAS4/ARNT2 bound to variant Fam-labeled dsDNA probes. **B.)** quantification of EMSA binding at submaximal (100ng) EMSA binding vs predicted Affinity from TF-DNA binding model. Mean (±SEM) of 3 independent experiments. **C.) (left panel)** Area under receiver operator curves (AUROC) (±SD) for the ability of NRLB DNA binding model for ARNT, ARNT2 or NPAS4 to predict NPAS4, NPAS4/ARNT, or NPAS4/ARNT2 rat ChIP-peaks vs size matched random sequences. NPAS4/ARNT2 TF-DNA binding model was used to score ChIP-peaks or randomly selected size matched regions to compare the ability to identify true positive transcription factor binding sites. **(right panel)** Receiver operator curves comparing the model-based prediction of NPAS4 occupied sites for rat NPAS4/ARNT2 rat ChIP-seq subset by peak score (i.e top 10% of sites = top 10% of peak scores). The AUROC is indicated on each corresponding line. **D.)** **(left panel)** Receiver operator curves comparing the model-based prediction of NPAS4 occupied sites for mouse NPAS4/ARNT2 ChIP-seq subset by peak score (i.e top 2000 or 5000 of sites). The AUROC is indicated on each corresponding line. **(right panel)** NPAS4 mouse ChIP-seq peaks were scored using a NPAS4/ARNT2 NRLB model, binned by motif affinity (1-10; low to high) and compared to average NPAS4 ChIP peak score (± sem). Linear regression p-value $p < 2 \times 10^{-16}$. **E.)** NPAS4 rat ChIP-seq peaks were scored using a ARNT (red), ARNT2 (blue) or NPAS4/ARNT2 (green) NRLB models, binned by motif affinity (1-10; low to high) and compared to average NPAS4, NPAS/ARNT or NPAS4/ARNT2 ChIP peak score (± sem). Linear regression p-value $p < 2 \times 10^{-16}$. **F.)** Area under receiver operator curves (±SD) for the ability of NRLB DNA binding models for ARNT (top left), HIF1α/ARNT (top right ; round 3,

random 18mer library), HIF2α/ARNT(bottom left ; round 3, random 18mer library), or HIF2α_r2/ARNT(bottom right); round 2, FixedCore 18/22mer library) to predict Tf (ARNT, HIF1a, HIF2a, HIF1a/HIF2a, HIF1a/ARNT, HIF2a/ARNT) ChIP-peaks from hypoxically treated HepG2 cells. NRLB models were used to score ChIP-peaks or randomly selected size matched regions to compare the ability to identify true positive transcription factor binding sites. **G.)** HIF1α or HIF2α ChIP-peak DNA (HepG2) was scored using NRLB Models for ARNT (red), HIF1a (light blue, round 3 18mer library), HIF2a (green, round 2 18/22mer fixed core library) or HIF2a (dark blue , round 3 18mer library), binned by motif affinity (1-10; low to high) and compared to average HIF1a (left) or HIF2a (right) ChIP peak score **(**mean ± SEM). **H.) (left panel)** Area under receiver operator curves for the ability of NRLB DNA binding models for HIFα to predict HIF1α or HIF2α ChIP-peaks from hypoxically treated MCF7 cells or randomly selected regions. **(right panel)** linear regression of binned affinity scores (1-10; low to high) vs mean ((±SEM) HIF1α or HIF2α ChIP peak scores p-value $p < 2 \times 10^{-16}$.

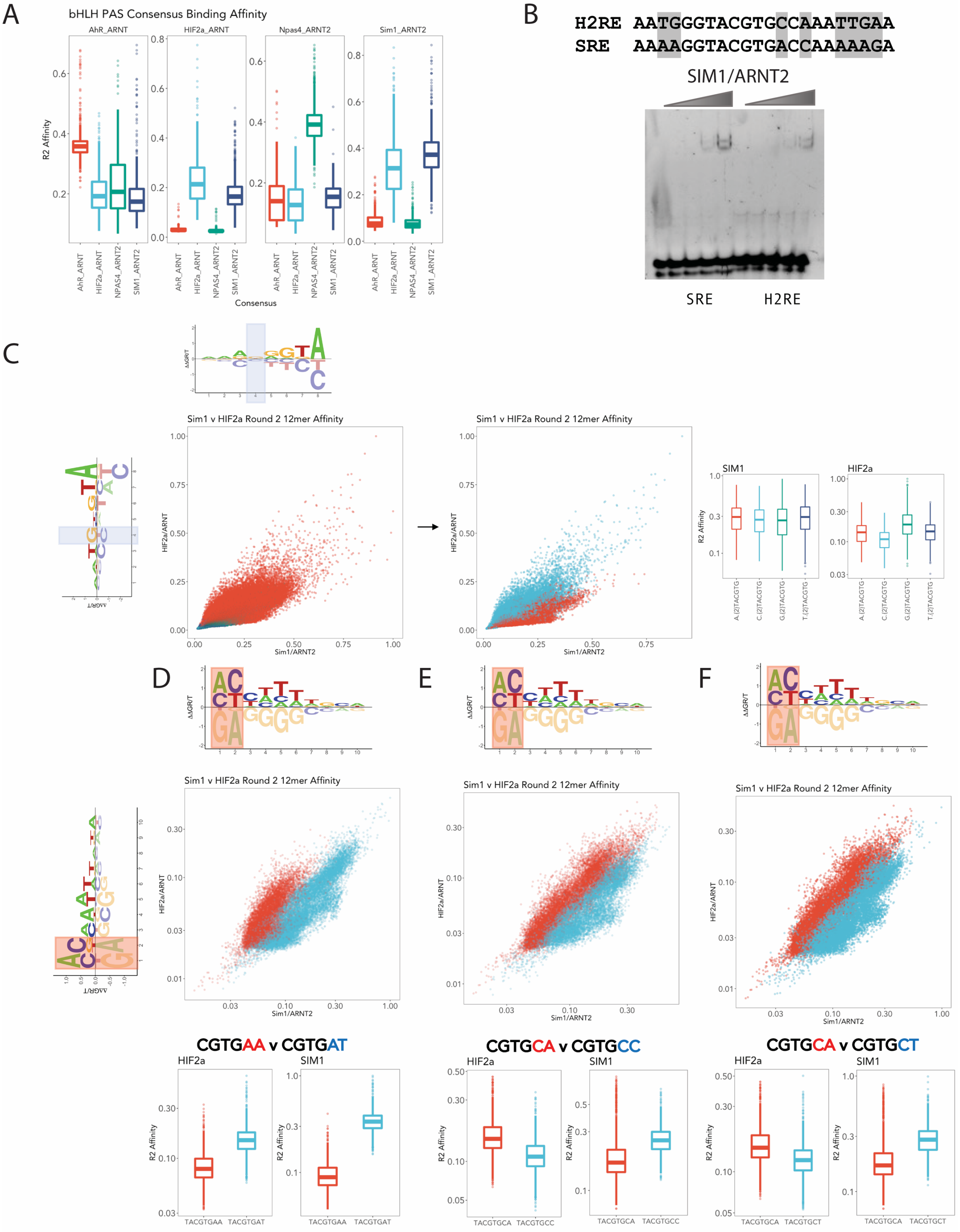

Supplementary Figure 6. Comparison of SIM1/ARNT2 v HIF2α/ARNT DNA binding specificity encoded in Core flanking sequences. A.) Boxplots of Kmer affinities for the bHLH-PAS transcription factors (panels left ➔ right) AhR/ARNT, HIF2α/ARNT, NPAS4/ARNT2 and SIM1/ARNT2 from the following consensus SIM1/ARNT2 consensus = GTACGTGMY, HIF2α_ARNT consensus = GNGTACGTGM, NPAS4_ARNT2 consensus = RRDRTCGTGAY, or AhR_ARNT consensus = TTGCGTGHC (IUPAC code; R = A or G, Y = C or T, M = A or C, D = A or G or T, H = A or C or T, N = any base.) **B.)** Top Kmer sequences from modelled DNA binding sites for SIM1/ARNT2 (Sim1 response element (SRE)) or HIF2a/ARNT (HIF2a response element (H2RE)) were used in EMSA gel shift assays to confirm SIM1/ARNT2 transcription factor preference for the SRE vs H2RE. shaded nucleotides indicates variant positions **C-F.)** Transcription factor specificity encoded through the upstream flank (shaded). **C.)** Upstream nucleotide preferences shown on NRLB energy logos for HIF2α/ARNT (y) and SIM1/ARNT2 (x), scatter plot of all 12mer Kmer Affinities coloured by NCGTG (GCGTG = green, ACGTG = red) **and** scatter plot of all 12mer Kmer Affinities containing either GxxxxCGTG blue or CxxxxCGTG in red. **Right panel.** Boxplot of 12mer Kmer NxxTACGTG affinities for SIM1/ARNT2 or HIF2α/ARNT. **D-E.)** Transcription factor specificity encoded through the downstream flank (shaded). Downstream nucleotide preferences shown on NRLB energy logos for HIF2α/ARNT (y) and SIM1/ARNT2 (x), scatter plot 12mer Kmer Affinities (log_{10}) selected for the presence of the indicated dinucleotide downstream. coloured by HIF2α preference (Red) or SIM1 preference (Blue). Boxplots of indicated comparisons lie below the scatterplots.

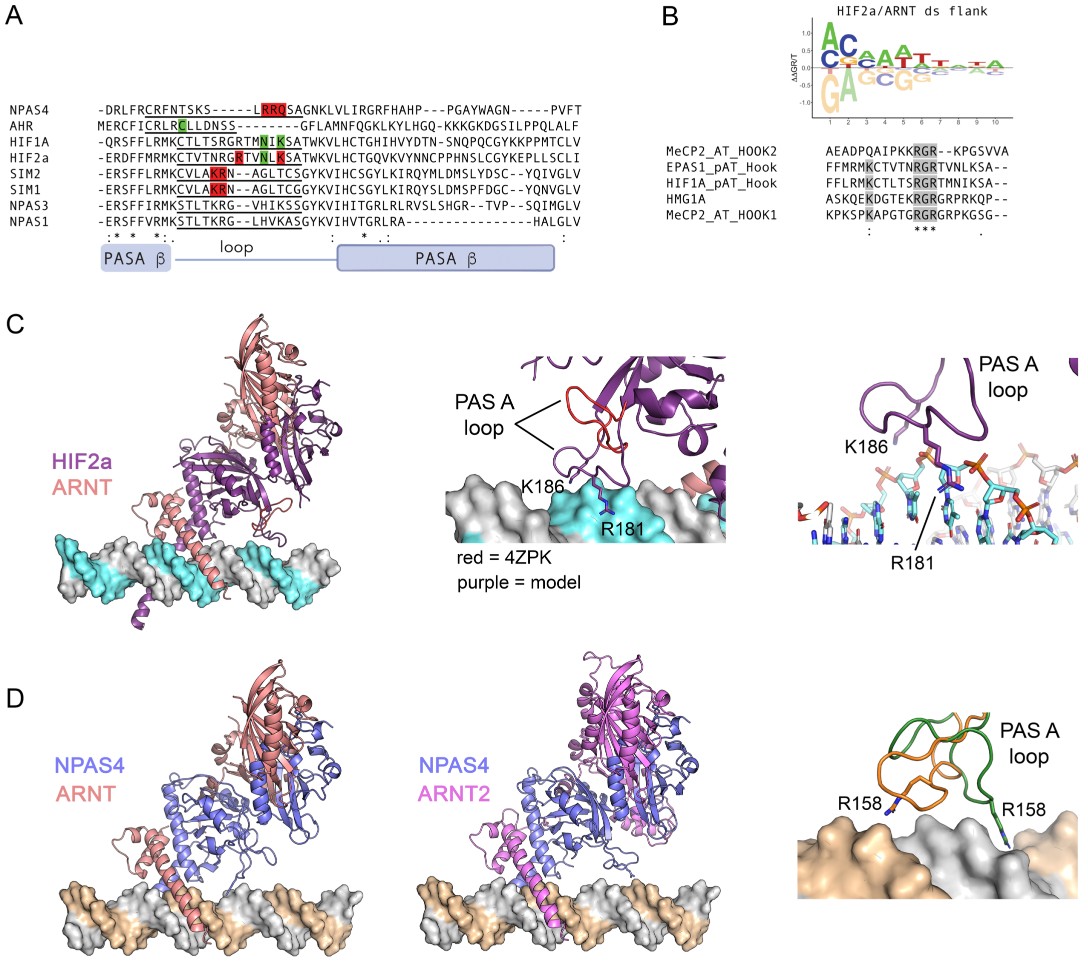

E

TOPMed PAS A Variants									
Protein	Variant	SNPloc	ALT allele count	ALT allele frequency	HET	HOMO	GnomAD	Phenotype	REF
NPAS4	R159S	11-66422718-C-A	1	0.000003778	1	0	No	unknown	
NPAS4	R158K	11-66422716-G-A	1	0.000003778	1	0	Yes	N/A	
EPAS1(HIF2a)	R181S	2-46360724-C-A	3	0.00113	3	0	No	unknown	
EPAS1(HIF2a)	R181C	2-46360724-C-T	3	0.00113	3	0	No	unknown	
EPAS1(HIF2a)	R181H	2-46360725-G-A	2	0.000756	2	0	No	unknown	
SIM1	R171H	6-100897270-G-A	3	0.0476 / 0.00	3	0	No	Obesity	Ramachandrappa et al, 2013

Supplementary Figure 7. PAS A loop DNA interaction mediated by Arginine residues. A.) Alignments of Class I bHLH-PAS A domains with basic residues in close proximity or contacting DNA from structures (or models) indicated in red and mutations leading to reduced DNA binding indicated in green. Loop regions extending from the main structure are underlined. **B**.) Alignment of PAS A loop from HIF1a and HIF2a with the AT-hook regions of MeCP2 (RGR motif is shaded). **C.)** HIF2a/ARNT/SRE model (based on crystal structure 4ZPK; HIF2a – purple, ARNT – pink) PAS loop remodeling vs crystal structure (4ZPK – red) shows the PAS loop rearrangement to more distally penetrate into the major groove at T16 and T17 (f_{+6} and f_{+7}), and AT rich regions identified by NRLB models) allowing R181 and K186 interaction with DNA. **D.)** Structural models of NPAS4/ARNT/NRE (left) or **D**.) NPAS4/ARNT2/NRE (right) show that the PAS loop can adopt multiple different conformation in which Arginine R158 residues come in close contact with DNA backbone downstream of the core DNA binding site **E.)** Human variants in bHLH-PAS transcription factors at DNA interacting arginine residues in the PAS-A loop. SIM1 R171H was previously identified as a variant associated with severe hyperphagic obesity.

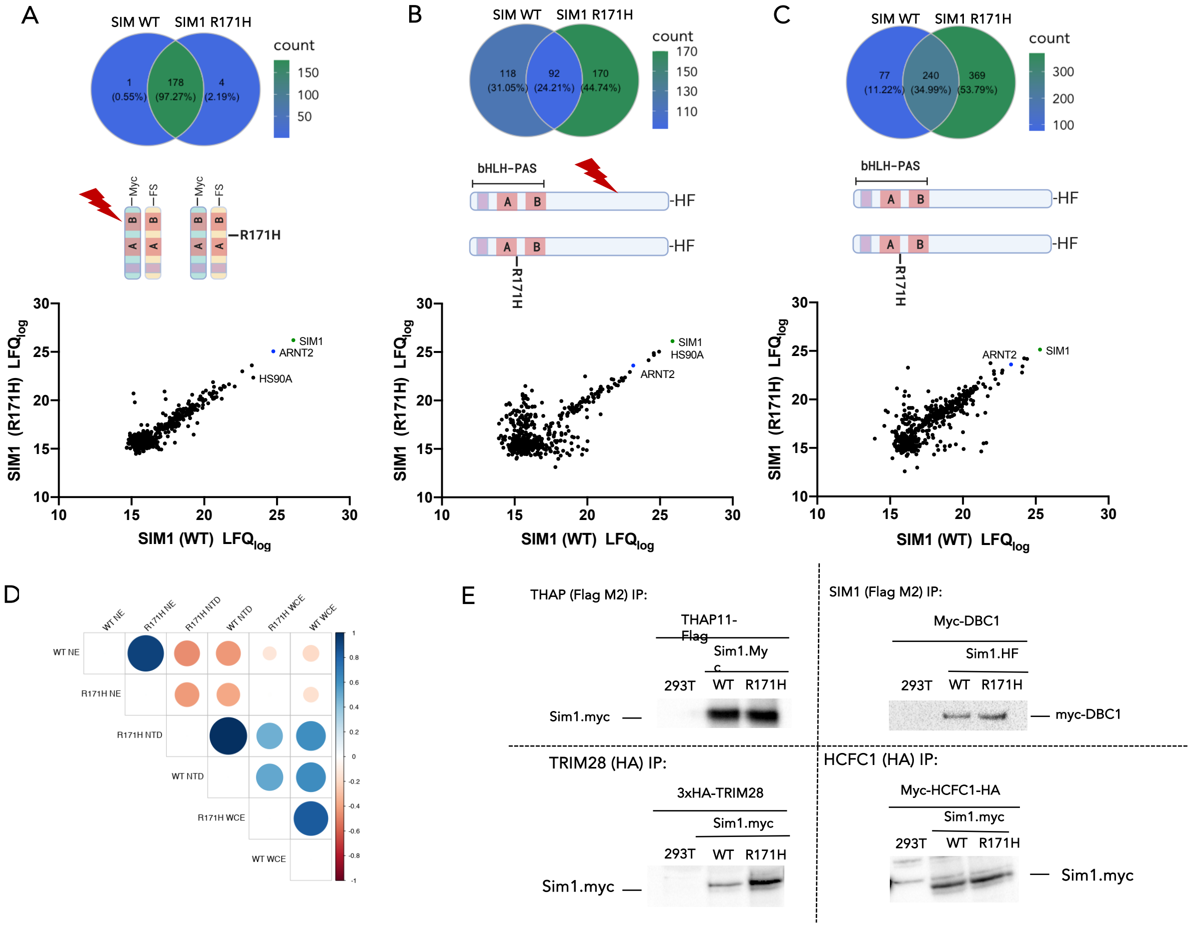

Supplementary Figure 8. SIM1 interaction proteomics reveals similar interactome profiles for SIM1 WT vs SIM1 R171H. Three separate strategies were used to investigate SIM1 interacting proteins and identify potentially preferential interaction with WT SIM1 A.) Co-expression of N-terminal SIM1.FS and ARNT2-Myc in HEK293T cells followed by DSP in cell crosslinking and immunopurification from whole cell lysates B.) stable SIM1.HF expression in HEK293T cells DSP crosslinking and immunopurification of SIM1 complexes from whole cell lysates C.) stable SIM1.HF expression in HEK293T cells, immunopurification of SIM1 complexes from nuclear extracts. A-C.) Upper panels show Venn diagram overlap of proteins identified by mass spectrometry proteomics and schematic of strategies used to isolate complexes. Lower panel is scatterplots of Log transformed lable free quantification (LFQ) of proteins identified by mass spectrometry. D.) Correlation matrix of LFQ from each of the interaction proteomics strategies. (NE = Nuclear Extract, NTD = N-Terminal Domain, WCE = Whole Cell Extract). E.) Co-immunoprecipitation of proteins identified in interaction proteomics. Indicated proteins were coexpressed in HEK293T and Co-IP's and western blots were performed to confirm interactions.

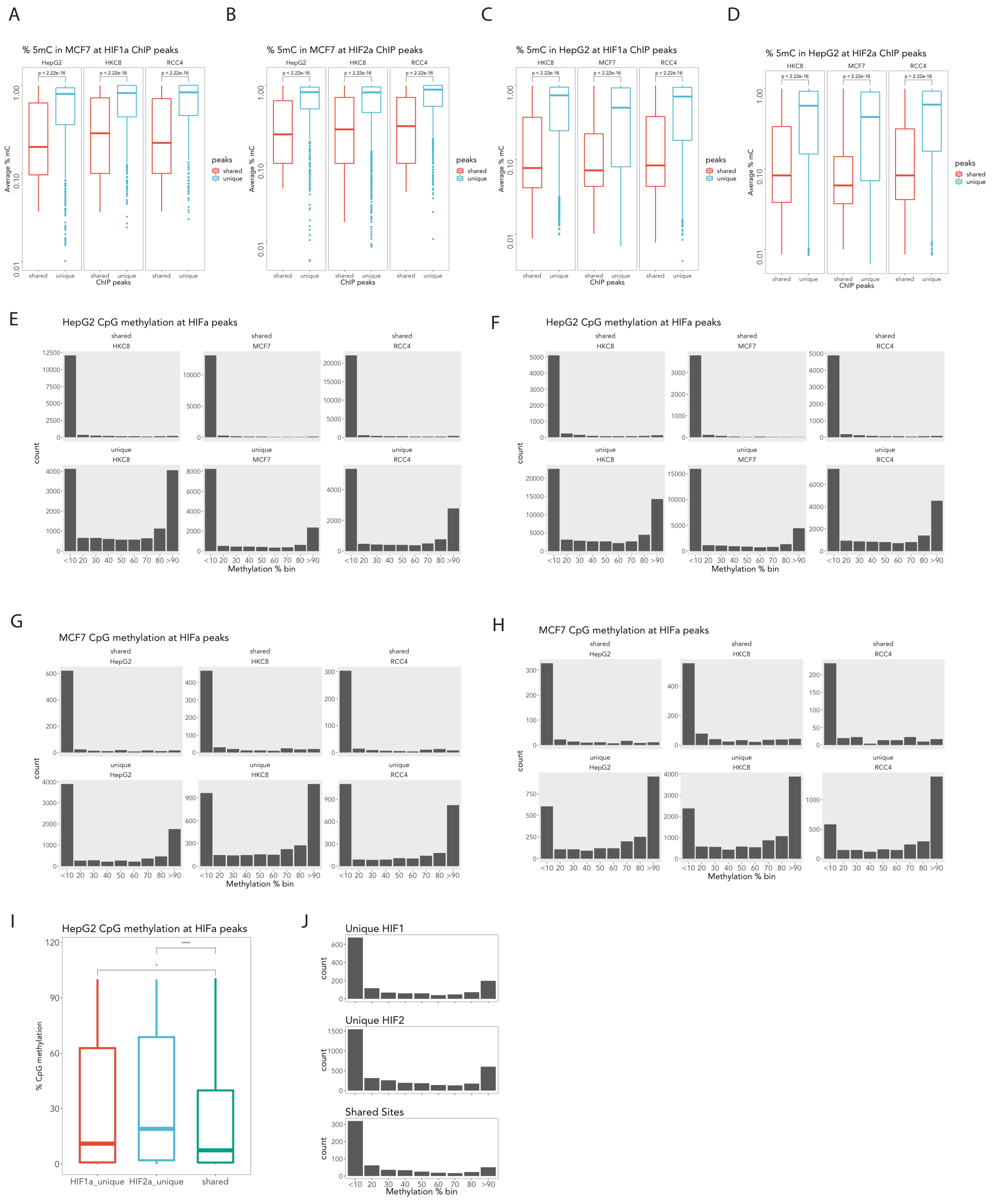

Supplementary Figure 9. Cell type specific methylation directs bHLH-PAS transcription factor occupancy. A.) and **B.)** Average % CpG methylation in MCF7 cells at **A.)** HIF1α or **B.)** HIF2α unique to HepG2, HKC8, or RCC4(vhl) ChIP peaks or shared HIFα MCF7 ChIP peaks. **C.)** and **D.)** Average % CpG methylation in HepG2 cells at **C.)** HIF1α or **D.)** HIF2α unique to HKC8, MCF7 or RCC4(vhl) ChIP peaks or shared HIFα HepG2 ChIP peaks. p-values represent an unpaired comparison of %CpG methylation at common or shared peaks vs unique peaks. **E.)** The number of HIF1a ChIP peaks at HepG2 methyl CpG % CpG methylation bins (0-100%) for shared HIF1α ChIP peaks shared between HepG2 and indicated cell lines (upper panel) or unique to the indicated cell lines (lower panel; MCF7, HKC8, and RCC4(vhl)) **F.)** The number of HIF2α ChIP peaks at HepG2 methyl CpG % CpG methylation bins (0-100%) for shared HIF2α ChIP peaks between HepG2 and indicated cell lines (upper panel) or unique to the indicated cell lines (lower panel; MCF7, HKC8, and RCC4(vhl)) **G.)** The number of HIF1a ChIP peaks at MCF7 methyl CpG % CpG methylation bins (0-100%) for shared HIF1a ChIP peaks shared betweenMCF7 and indicated cell lines (upper panel) or unique to the indicated cell lines (lower panel; HKC8, HepG2, and RCC4(vhl)) **H.)** The number of HIF2a ChIP peaks at MCF7 methyl CpG % CpG methylation bins (0-100%) for shared HIF2α ChIP peaks between MCF7 and indicated cell lines (upper panel) or unique to the indicated cell lines (lower panel; HKC8, HepG2, and RCC4(vhl)). **I.)** Average percentage HepG2 CpG methylation at HepG2 HIFa ChIP peaks (HIF1α unique peaks (red), HIF2a unique peaks (blue), HIF1α/HIF2α shared

peaks (green). * p-value < 1×10^{-6} and *** p-value < 1×10^{-10}. **J.)** number of HIFα ChIP peaks in % CpG methylation bins (0-100%), HIF1a unique peaks (upper panel), HIF2a unique peaks (middle panel), or HIF1a/HIF2a shared peaks (lower panel).
